# Development of a 76k Alpaca (*Vicugna pacos*) Single Nucleotide Polymorphisms (SNPs) Microarray

**DOI:** 10.3390/genes12020291

**Published:** 2021-02-19

**Authors:** Marcos Calderon, Manuel J. More, Gustavo A. Gutierrez, Federico Abel Ponce de León

**Affiliations:** 1 Facultad de Zootecnia, Universidad Nacional Agraria La Molina, Lima 15024, Peru; mcalderon.montes@gmail.com (M.C.); mmoremontoya@gmail.com (M.J.M.); gustavogr@lamolina.edu.pe (G.A.G.); 2 Escuela de Formación Profesional de Zootecnia, Facultad de Ciencias Agropecuarias, Universidad Nacional Daniel Alcídes Carrión, Cerro de Pasco 19001, Peru; 3 Department of Animal Science, University of Minnesota, Minneapolis, MN 55108, USA

**Keywords:** single nucleotide polymorphisms (SNPs), microarrays, alpaca, *Vicugna pacos*, fiber genes

## Abstract

Small farm producers’ sustenance depends on their alpaca herds and the production of fiber. Genetic improvement of fiber characteristics would increase their economic benefits and quality of life. The incorporation of molecular marker technology could overcome current limitations for the implementation of genetic improvement programs. Hence, the aim of this project was the generation of an alpaca single nucleotide polymorphism (SNP) microarray. A sample of 150 Huacaya alpacas from four farms, two each in Puno and Cerro de Pasco were used for SNP discovery by genotyping by sequencing (GBS). Reduced representation libraries, two per animal, were produced after DNA digestion with ApeK1 and double digestion with Pst1-Msp1. Ten alpaca genomes, sequenced at depths between 12× to 30×, and the VicPac3.1 reference genome were used for read alignments. Bioinformatics analysis discovered 76,508 SNPs included in the microarray. Candidate genes SNPs (302) for fiber quality and color are also included. The microarray SNPs cover 90.5% of the genome length with a density of about 39 ± 2.51 SNPs/Mb of DNA at an average interval of 26.45 ± 18.57 kbp. The performance was evaluated by genotyping 30 family trios and comparing them to their pedigrees, as well as comparing microarray to GBS genotypes. Concordance values of 0.93 and 0.94 for ApeK1 and Pst1-Msp1 generated SNPs were observed. Similarly, 290 fiber quality and color candidate gene SNPs were validated. Availability of this microarray will facilitate genome-wide association studies, marker-assisted selection and, in time, genomic selection.

## 1. Introduction

The population of alpacas in Peru is about 3,685,516 [1], representing 85% of the world population of this domestic species. Alpacas are specialized in fiber production, placing Peru as the top world producer and exporter of this product for the textile industry [2]. Genetic improvement of fiber production and fiber quality characteristics could contribute to increased economic benefits and better quality of life for producers. However, several challenges remain to be overcome for the implementation of genetic improvement programs. To name a few, dispersion of small producers rearing about 90% of the alpaca population under extensive management conditions without reproductive management implemented, and no production records kept. Hence, genetic improvement programs at the small producer level are nonexistent and, at best, are done by casually acquiring quality sires at agricultural fairs. Some of these challenges can be resolved with the development and implementation of new technologies coupled with extension educational programs. At present artificial insemination technology, with diluted semen, is available, allowing, in comparison to natural mating, some marginal increase in the use of selected sires [3,4]. The development of long-term semen preservation technology will expand the use of selected sires. Similarly, the development of a single nucleotide polymorphism (SNP) microarray would facilitate the implementation of genetic improvement programs centered on the use of marker-assisted selection and genomic selection as alternatives to increasing genetic progress, the latter by shortening generation interval(s).

Several advances in understanding the organization of the alpaca genome have occurred in the last decade. The alpaca genome is sequenced and assembled into 77,389 scaffolds (VicPac3.1, GCA_000164845.4, NCBI), of which 88 scaffolds are assigned to chromosomes and represent 76% of the reference genome length [5]. The rest of the genome (24%), represented by 77,301 scaffolds, is not assigned to chromosomes. Chromosomal syntenies among camelids, bovine and humans have been identified by Zoo-FISH [6], and several genes and molecular markers are assigned to chromosomes by fluorescence in situ hybridization (FISH), allowing the development of the first cytogenetic map [7], which has recently been expanded [8,9]. In addition, single nucleotide polymorphisms (SNPs) have been identified in alpacas using a high-density bovine SNP microarray, allowing the identification of 6756 conserved SNPs between bovine and alpaca [10]. A small number (63) of SNPs and nucleotide mutations found in candidate genes for fiber quality and color have also been reported [11,12,13,14,15,16]. SNP microarrays have been developed for several domestic species. In each case, researchers aimed at identifying large numbers of highly informative SNPs localized at equidistant intervals along the genome. The 50 K bovine microarray [17] is based on novel SNPs specifically developed for the microarray and a collection of SNPs reported in the literature and available in databases. Novel SNPs were identified after sequencing and aligning sequencing reads from three reduced representation DNA libraries (RRLs) made from three pools of DNA. One pool was from 15 Holstein cows, the second pool from 35 Angus bulls and the third pool from two bulls of each of the Charolais, Gelbvieh, Hereford, Limousin, Red Angus and Simmental breeds. The reason for using these two DNA breed pools and one mixed breed DNA pool was to enhance the probability of identifying sequence variants among animals within a breed and among breeds. A similar approach was also used for the development of the 60 K SNP porcine microarray [18], which was based on 19 RRLs generated from four swine breeds (Duroc, Landrace, Large White, Piétrain) and a Wild Board population comprising a total of 158 animal samples originating from the USA, the Netherlands, Denmark, Europe and Japan. This strategy identified 372 K novel SNPs, of which 64 K were selected for inclusion in the microarray. Again, animal sampling of several breeds enhanced the probability of identifying DNA sequence variants and the utility of the microarray.

Similarly, the chicken 60 K SNP microarray [19] used 25 DNA samples obtained from each of 4 lines of commercial breeding birds, two meat-type and two egg-type lines. Researchers generated four reduced representation libraries with DNA pools from each bird line. After sequencing and filtering, they identified 561 K SNPs, of which they selected 61 K SNPs for inclusion in the microarray. Even though the sampling of birds was constrained to only four breeding lines, researchers were able to identify a large number of SNPs.

Our aim is the construction of an alpaca SNP microarray to facilitate future alpaca genetic studies and the implementation of selection programs for fiber diameter and other production traits. Our preference for generating an alpaca microarray over the use of genotyping by sequencing (GBS) genotyping is based on the practicality of the microarray used by small producers in general and across research institutions in particular. The use of a microarray would allow comparability of data generated by independent studies, thus magnifying genetic information of the species and allowing future meta-analysis. The inherent pros and cons for SNP microarray and GBS genotyping are not the scope of this study. However, we consider it important to indicate that microarrays are prepared to cover genomes at more or less fixed intervals and are designed to include SNPs with MAFs ≥ 0.05, therefore, avoiding rare SNPs [20]. The GBS methodology generates SNPs randomly located across the genome and generates rare SNPs with higher frequency. GBS accuracy in reading heterozygote genotypes depends on the read depth of SNPs per population study, therefore, requiring more bioinformatics analysis than SNP microarrays. GBS, however, has the advantage of being more cost-effective than microarray genotyping. Recently, Fan et al. [21] have successfully confirmed evolutionary relationships among South American camelids based on GBS. In general, the use of one method over the other depends on the objectives of each study and/or the objectives of long-term collaborative programs.

The use of an alpaca microarray for genome-wide association studies (GWAS) between markers and production traits would help in the development of alpaca genetic improvement programs based on marker-assisted selection and, in due time, genomic selection.

## 2. Materials and Methods

### 2.1. Ethics Statement

Alpaca blood samples for DNA isolation were obtained in accordance with the Peruvian National Law No. 30407, “Animal Protection and Welfare Law”, in effect in Peru since 7 January 2016. The Dean of the College of Animal Science approved the protocol in lieu of the UNALM “Ethics Committee for Scientific Research” No. 0345-2018-CU-UNALM—October 22, 2018, that has not started operations.

### 2.2. Selection of Animals and DNA Sequencing

Several criteria were established for the identification of 150 Huacaya alpacas to make up the sample used for the identification of single nucleotide polymorphisms (SNPs). The sampled animals originate from at least two different Andean geographic regions (Puno and Pasco) and two farms per region. Pedigree and production records at second shearing for each animal were available. Animals were healthy and reared under an adequate extensive management system. The number of animals by region and farm within the region for the collection of blood samples is shown in Table 1.

Blood samples (6 mL) were collected in vacutainer tubes containing 10.8 mg of EDTA-K_2_. DNA extraction was performed using the PureLink™ kit (Invitrogen, CA, United States). DNA quality was evaluated by agarose gel electrophoresis (0.8%) at 120 volts in 1× TBE buffer for 30 min. Samples were lyophilized in a concentrator plus (Eppendorf, Hamburg, Germany) in 0.6 mL microcentrifuge tubes at a temperature of 45 °C for 90 minutes. Samples were outsourced to AgResearch Limited (Hamilton, New Zealand) for the preparation of 300 reduced representation libraries (RRLs) and DNA sequencing. DNA digested with ApeK1 and double digested with Pstl/Mspl were prepared for each animal sample. DNA fragment sizes between 100 and 200 bp were selected for library preparations (two RRLs per animal). DNA sequencing was done with the HiSeq 2500 sequencer at an average reading depth of ~6× per library.

### 2.3. Identification and Selection of SNPs

The ApeK1 and Pstl-Mspl 300 RRLs generated reads were subject to quality control using FastQC software (https://www.bioinformatics.babraham.ac.uk/projects/fastqc/). Reads exceeding the Illumina quality score threshold of 20 for sequence quality were used to discover reliable SNPs [18]. Quality controlled reads were aligned to the VicPac3.1 reference genome (NCBI, GCA_000164845.4). Burrows–Wheeler aligner (BWA) software and SamTools were used for reference genome indexing [22,23]. Picard tools were used for “dictionary” sequence creation, sorting, duplicate marking, indexing and file merging [24]. BWA and BCFtools were used for reading alignment and for variant identification and calling, respectively [25].

The procedures for the selection of the SNPs were executed using scripts programmed in R (File S1). Only SNPs with Phred scale >10, genotyping rate (GR) ≥ 0.15, minor allele frequency (MAF) between 0.01 and 0.50, Illumina design score ≥ 0.6 (Design Studio—Microarray Assay Designer software. Illumina, CA, USA) and absence of other SNPs within the flanking sequences were considered for possible inclusion in the microarray. Quality scores for the first round of selection were set at Phred scale > 10, GR ≥ 0.45, MAF ≥0.05 and Illumina design score ≥0.6. For subsequent rounds of selection, only GR and MAF were decreased to ≥0.15 and ≥0.01, respectively.

To distribute selected SNPs along the genome at 40 kbp intervals, a 40 kbp location score was used. The following, adapted formula [17] was applied:

$$\mathrm{Location}\;\mathrm{score}=\;\left(E—S\right)\;-\;\left(2a\;-\;\left(E\;+\;S\right)\right)$$

E—the final position of the fragment;

S—the initial position of the fragment;

a—the position of the SNP.

A location score was defined for each equidistant interval of 40 kbp along each scaffold. The location score was calculated according to the position of the SNP within each 40 kbp fragment. SNPs with a high score are located at or near the middle position of the 40 kbp fragment, and SNPs with a low score are located far from the middle position and near the ends of the fragment. An example of the application of the location score for 40 kbp fragments is provided in Table S1. With the objective to increase the density of SNPs for the microarray, a second SNP was selected within each 40 kbp fragment. To that end, each 40 kbp fragment was divided into five 8 kbp subfragments, coded as subfragment 1 (from 0 to 8 kbp), subfragment 2 (from 8 to 16 kbp), up to subfragment 5 (from 32 to 40 kbp). After this, the 8 kbp subfragment containing the first selected SNP was identified. Based on which subfragment contained the first selected SNP, a second SNP was selected in another subfragment as follows: If the first SNP was located in subfragment 1, the second SNP was selected for subfragment 3. If the first SNP was located in subfragment 2, the second SNP was identified for subfragment 4. In this manner, the second SNPs were always identified two subfragments down in a clockwise direction for each 40 kbp fragment. This selection procedure was done with an R script [26] (File S1). Hence, for the construction of the alpaca SNP microarray, two sets of SNPs were generated. The first set identified SNPs spaced at 40 kbp intervals across the genome, and the second set increased the density and reduced the average spacing between SNPs. To calculate the chromosomal length in base pairs covered with SNPs at fixed intervals, we added the length in base pairs of all the fragments containing an SNP per scaffold and per chromosome. A similar process was done for all scaffolds not assigned to chromosomes. The percent covered was calculated based on chromosome and genome length as reported for the VicPac3.1 reference assembly.

In addition, the third set of SNPs located at or nearby candidate genes for fiber quality and color (*KRT, KRTAP, ASIP, MC1R, TYRP1*) as annotated in the VicPac3.1 reference genome was also included. To identify this third set of SNPs sequence reads from ten Huacaya alpaca genomes sequenced at an approximate depth of 30×, (NCBI Accession codes SRR13340600 to SRR13340605 and SRX2065156 to SRX682159) and the 300 RRLs were aligned to the VicPac3.1 reference genome. Only SNPs with Illumina score ≥0.60 and devoid of other SNPs in their flanking sequences were selected.

The list of the three sets of SNPs was submitted to NEOGEN (Lincoln, NE, USA) for further evaluation to identify the final set of SNPs for the microarray-based on Affymetrix (CA, USA) SNP evaluation algorithms.

### 2.4. Performance of the Alpaca SNP Microarray

Thirty (10 from Pasco and 20 from Puno) trios (sire, dam and progeny) were identified based on their pedigrees and were genotyped with the generated alpaca SNP microarray. Genotypes of 68 animals that make up the 30 alpaca trios were analyzed using the Axiom Analysis Suite software (Affymetrix, CA, USA).

For the calculation of the matrix of genomic relationships (G), SNP genotypes, monomorphic SNPs and SNPs with low rates of genotyping were removed. SNP genotypes were coded as 0, 1 and 2 for AA, AB and BB, respectively, where A is the reference allele and B is the alternate allele. GCTA [27] was used for the calculation of the matrix of relationships (G), where the relationship between individual *j* and *k* is estimated with the following equation:

(1) $$G_{jk}=\;\frac{1}{N}\;\overset{N}{\underset{i=1}{\sum }}\frac{\left(x_{ij}-\;2p_{i}\right)\left(x_{ik}-\;2p_{i}\right)}{2p_{i}\;\left(1-\;p_{i}\right)}$$

*x_i_* = number of copies of the reference allele for the *ith* SNP;

*x_ij_* = number of copies of the refrence allele for the *ith* SNP of the *jth* individual;

*p_i_* = Frequency of the reference allele;

*N* = Number of SNPs;

Genotyping results were compared to pedigrees.

### 2.5. Comparison of Genotyping by Sequencing (GBS) and Microarray Genotyping (MG)

To assess the reliability of GBS and MG, genotypes were quality control evaluated. For the GBS genotypes, SNPs with less than three reads were manually (Microsoft Excel) removed from further analysis. Quality control of MG genotypes was done with the Axiom Analysis Suite v.4.0.3 software (Affymetrix, CA, USA). Sample quality control included the following parameters: dish QC (DQC) ≥0.82, QC call rate ≥97%, percent of samples passed DQC and QC call rate by plate ≥95% and average call rate for passing samples ≥98.5. The SNP quality control included a call rate ≥of 97% and the best and recommended SNPs (poly-high-resolution, no-minor-hom, mono-high-resolution). Only 145 of 150 MG genotypes passed the quality control.

Each SNP genotype in the GBS and MG datasets was coded 0, 1 and 2 for AA, AB and BB, where A represents the reference allele and B the alternate allele. The final database contained 75,577 SNPs (57,697 SNPs identified from the ApeK1 and 17,880 SNPs from the Pstl-Mspl RRLs) that are common to both datasets. The filtered GBS genotypes were compared, pairwise, to MG genotypes, per animal to determine the level of concordance between both genotyping methods. Comparisons were done using BCFtools. Each comparison was expressed as the score concordance between both genotyping methods.

### 2.6. Animal Sample Population Structure

To assess the population structure of the animal sample, we used genotyping results obtained by GBS and MG separately. For both genotyping methods, we calculated the genomic relationship matrix as previously described taking into consideration SNPs with call rate ≥ 0.9 and MAF ≥ 0.05. The principal component analysis was used to infer population differentiation [28,29]. The heat map of genomic relationships was generated with the software RStudio Team [30] (File S2).

The inbreeding coefficient and individual heterozygosity for microarray genotyping were calculated using data of 145 alpacas. SNPs located on the X chromosome as well as SNPs with call rates < 0.90 and MAF < 0.05 were excluded. After linkage disequilibrium-based SNP pruning, 29,059 variants were retained for the analysis.

For the calculation of the coefficient of heterozygosity and coefficient of inbreeding with GBS data, 2,452,915 ApeK1 and 884,297 Mspl-Pstl SNPs were used. A minimum of three sequencing reads, per allele, per SNP and per animal, were required per GBS genotype call. The coefficient of individual heterozygosity was calculated with PLINK v1.90p and represents the proportion of heterozygote loci of the total genotyped loci per animal sample [31]. The coefficient of inbreeding (*F*) was calculated with GCTA [27], where the estimate is based on the correlation between gamete bindings:

$${\hat{F}}_{i}^{III}=\;\left[x_{i}^{2}-\left(1+2p_{i}\right)x_{i}+2p_{i}^{2}\right]/h_{i}\;\;y\;var\;({\hat{F}}_{i}^{III}\left|F)\right.$$

$${\hat{F}}_{i}^{III}=\;1+2\left(1-2h_{i}\right)F/h_{i}-\;F^{2}$$

${\hat{F}}_{i}^{III}\;$ is an unbiased estimator of *F* in the sense that $E({\hat{F}}_{i}^{III}\left|F)=F\right.$. For multiple SNPs, it averages the estimations over all SNPs or:

(2) $$\hat{F}=\;1/N\sum_{i=1}^{N}{\hat{F}}_{i}$$

*x_i_* = number of copies of reference allele for the ith SNP;

*h_i_* = 2 *p_i_* (1 − *p_i_* );

*p_i_* = frequency of the reference allele;

*N* = number of SNPs.

To estimate the admixture of the four animal sample groups, we used the software program ADMIXTURE [32,33,34]. This program estimates the ancestry of unrelated individuals and provides a measure of admixture among populations. For the admixture analysis, we used the SNP microarray generated genotypes for the sample animals.

## 3. Results

### 3.1. Sequencing of Reduced Representation Libraries

The ApeK1 and Pstl-Mspl 300 RRLs generated reads were subject to quality control using the FastQC software (https://www.bioinformatics.babraham.ac.uk/projects/fastqc/, accessed on 18 October 2020). Reads (3.45 × 10^8^) exceeding the threshold of 20 for sequence quality were used to discover reliable SNPs [18].

Bioinformatics analysis to identify nucleotide variants by the alignment of quality reads from the 300 RRLs to the VicPac3.1 reference genome identified 3,427,450 and 1,297,789 variants from the ApeKI and the Pstl-Mspl RRLs, respectively. After eliminating one copy of any duplicate SNP, 4,283,956 were unique.

### 3.2. Selection of SNPs for the Microarray

Use of the quality score parameters for the selection of reliable SNPs reduced the original set of unique SNPs to 513,467. From these, 45,156 SNPs distributed at approximately 40 kbp intervals were selected (Table 2, round 1). To increase the number of 40 kbp fragments containing an SNP, other SNPs were selected in subsequent rounds (Table 2, rounds 2–6), generating 51,772 selected SNPs as our first set of SNPs for the microarray. To increase the density of SNPs, the second set of 28,429 SNPs was identified (Table 2). These two sets represent 80,201 novel alpaca SNPs (accession code: ERZ1694265).

Additionally, a set of exonic and intronic SNPs located in candidate genes for fiber quality and color include 302 SNPs (Accession code: ERZ1694264) as follows: *KRT*s (205), *KRTAP*s (56), *MC1R* (1), *ASIP* (2), *KIT* (18), *TYRP1* (1). Similarly, SNPs reported in the literature for *KRT*s (16) [15], *MC1R* (1) [13], and *TYRP1* (2) [14], are included.

All chromosomally assigned scaffolds, representing about 76% of the genome, contain at least one SNP per every 25.92 ± 14.57 kbp with a chromosome density of 40 SNPs/Mb (Figure S1). Unlocalized scaffolds, representing about 24% of the genome, on average, contain an SNP per every 23.69 ± 24.58 kbp. Only 78.2% of the total length of unlocalized scaffolds have assigned SNPs (Table S2). In this manner, we calculated that 92% of the total length of the genome contains one SNP per average DNA fragment of 25.41 ± 17.41 kbp. The distribution of SNPs by MAF is shown in Figure S2. SNPs with MAFs ≥ 0.10 represent 71.9% of the total.

### 3.3. Construction of the Alpaca SNPs Microarray

The constructed microarray includes 76,508 SNPs, with SNPs covering 95.2% of the length, in base pairs, of chromosomally assigned scaffolds and 76.1% of the unassigned scaffolds representing 90.5% of the length of the alpaca genome covered by SNPs at an average distance of 26.58 ± 18.57 kbp from each other (Table S3). Another 302 SNPs located at or near the coding sequences of candidate genes for fiber quality and color are also included in the SNPs microarray (Table S8). Table 3 presents the number of scaffold fragments containing one SNP. The largest number of SNPs (58,110) are positioned at distances between 10 to 30 kbp of each other along the genome.

In Table 4, we present the number of SNPs submitted to NEOGEN (Lincoln, NE, USA) and the final number of SNPs selected for inclusion in the microarray with standard Affymetrix (CA, USA) controls and duplicate SNPs controls. The SNP microarray thus includes 76,508 unique novel alpaca SNPs plus 100 Affymetrix controls and 302 duplicate SNP controls. The distribution of SNPs is presented in Table S3.

**Figure 1:**
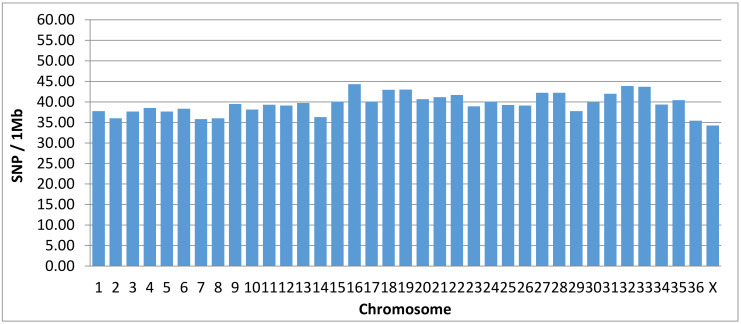
Density of SNPs per chromosome.

All chromosomally assigned scaffolds, representing about 76% of the alpaca genome, have 95.2% of its length covered, on average, with at least one SNP per 26.99 ± 15.75 kbp. The average chromosome SNP density is 39 ± 2.51 SNPs/Mb (Figure 1). The unallocated scaffolds represent about 24% of the genome and have, on average, 76.1% of their total length covered with at least one SNP per 25.15 ± 25.99 kbp. Hence, 90.5% of the 2.1 × 10^9^ base pairs of the VicPac 3.1 reference genome contains, on average, one SNP per every 26.58 ± 18.57 kbp represented in the alpaca microarray. (Table 5).

### 3.4. Performance of the Alpaca SNP Microarray

#### 3.4.1. Concordance between Pedigree and Microarray Genotyping for Trios

Sixty-eight animals that makeup 30 alpaca trios (sire, dam and progeny) were genotyped to evaluate the reliability of the microarray in assessing paternity. Of the 76,508 SNPs, 68,700 SNPs passed the genotype quality control (Axiom Analysis Suite, Affymetrix, CA, USA). After the removal of monomorphic and low MAF SNPs, 61,086 SNPs were used for the construction of the genomic relationship matrix. Genomic relationships among members of each evaluated trio are presented in Table S4.

Twenty-six pedigree-recorded trios were concordant with their calculated genomic relationships (G) matrix. Four sire–progeny pairs and one dam–progeny pair G values were not concordant with their pedigree recorded information. According to G values, one progeny out of 30 showed to have been assigned the wrong sire and dam. Among trios, sire half-sibs observed G values were in agreement with sire progeny G values. Table S5. If progeny were wrongly assigned to a sire, the putative half-sibs were not confirmed by their G values.

#### 3.4.2. Comparison between GBS and Microarray Genotyping

Microarray genotyping (MG) of the 150 animals from which the SNPs were originally developed was done to assess the validity of the novel SNPs generated by GBS. Five samples were excluded from further analysis after genotyping quality control (Axiom Analysis Suite, Affymetrix CA, USA). For the comparison of genotyping results between the GBS and MG methods, we used 76,508 SNPs. Genotype comparisons between GBS and MG methods included 57,687 ApeK1 and 17,880 Pst1-Msp1 SNPs. Genotyping concordance between microarray and GBS ApeK1 SNPs was 0.93, and between MG and GBS, Mspl-Pstl SNPs was 0.94. Results are presented in Tables S6 and S7, respectively.

### 3.5. Sample Population Structure

**Figure 2:**
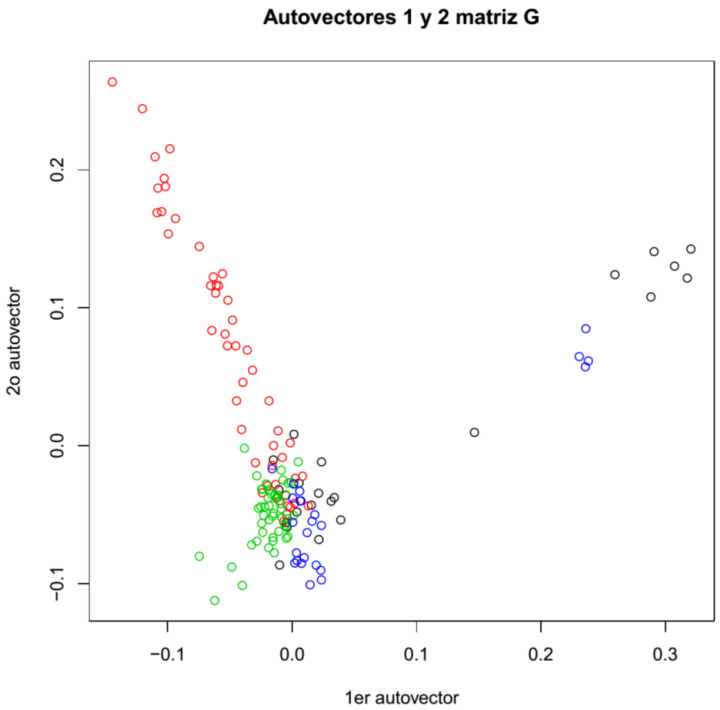
Population structure based on microarray genotyping (Pacomarca—red, Quimsachata—green, Racco—blue and Gacocen—black).

Both genotyping methods show equivalent population distributions of the sampled animals (Figure 2, Figures S3 and S4). Animals from Puno (Pacomarca and Quimsachata) present small overlapping. However, a portion of the animals from Cerro de Pasco (Gacocen and Racco) shows the most overlapping with animals from Quimsachata. The most differentiated group of animals was the Pacomarca group and a portion of the Racco animals. Quimsachata shows more differentiation from Pacomarca than from the other farms.

**Figure 3:**
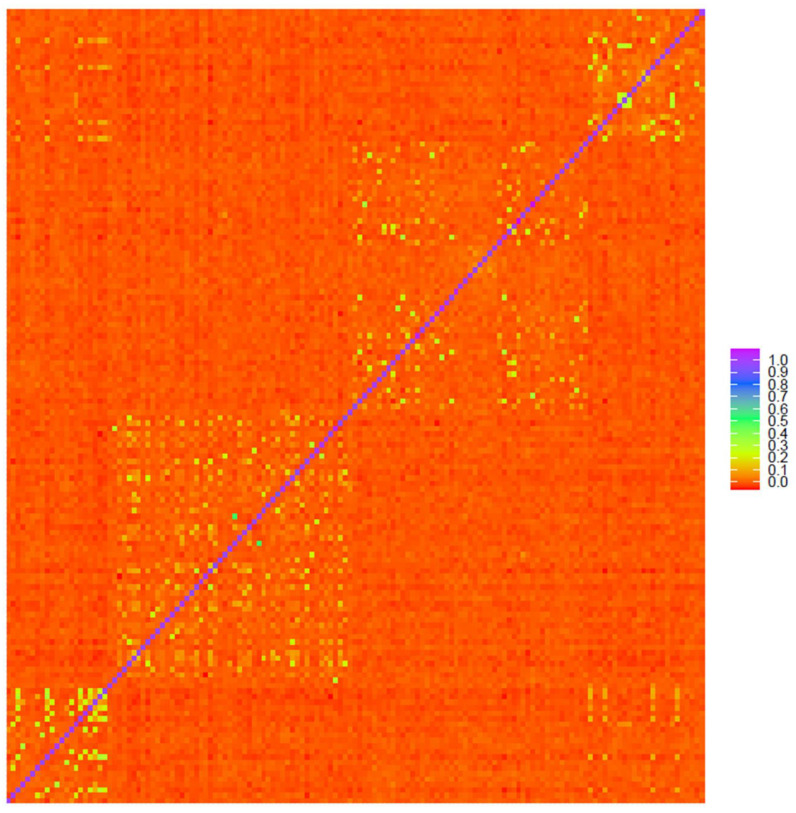
Heat map of genomic relationships among animals based on microarray genotyping Gacocen (bottom left), Pacomarca and Quimsachata (center area) and Racco (top right).

The heat maps of genomic relationships (Figure 3, Figures S5 and S6) show on the diagonal line four subpopulation blocks Gacocen (bottom left), Pacomarca and Quimsachata (center area) and Racco (top right), where it is clearly visualized that Gacocen and Racco showed more animals with common ancestors within and between farms. Pacomarca and Quimsachata showed ancestry that is more common within a farm but not between farms. The proportion of animals with 0.2 to 0.35 genomic relationships is 11.23% for Gacocen, 5% for Racco, 1.45% for Quimsachata and 0.82% for Pacomarca.

**Figure 4:**
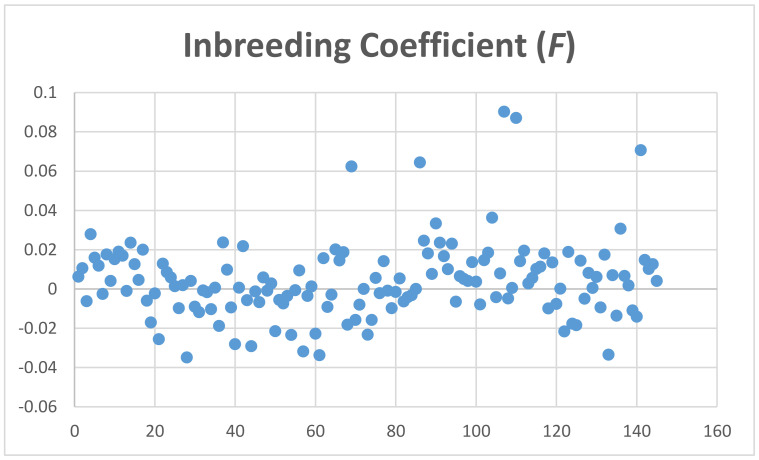
Inbreeding coefficient (F) of alpacas genotyped by microarray.

**Figure 5:**
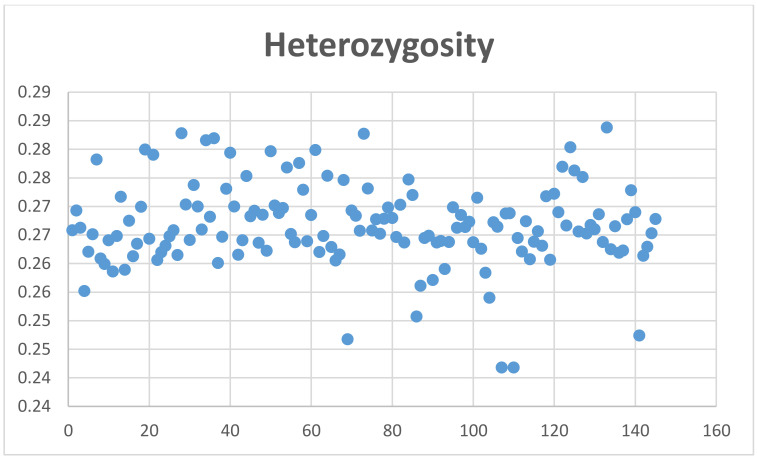
Level of heterozygosity of alpacas genotyped by microarray.

The average coefficient of inbreeding (F) calculated with the MG data was 0.004 ± 0.02, and using GBS genotyping for Mspl-Pstl and ApeK1 SNPs was 0.1060 ± 0.033 and 0.158 ± 0.018, respectively (Figure 4, Figures S7 and S8). The average coefficient of individual heterozygosity for microarray genotyping was 0.27 ± 0.01, and for GBS genotyping for Mspl-Pstl, and ApeK1 SNPs was 0.1327 ± 0.037 and 0.0947 ± 0.0225, respectively (Figure 5, Figures S9 and S10).

**Figure 6:**
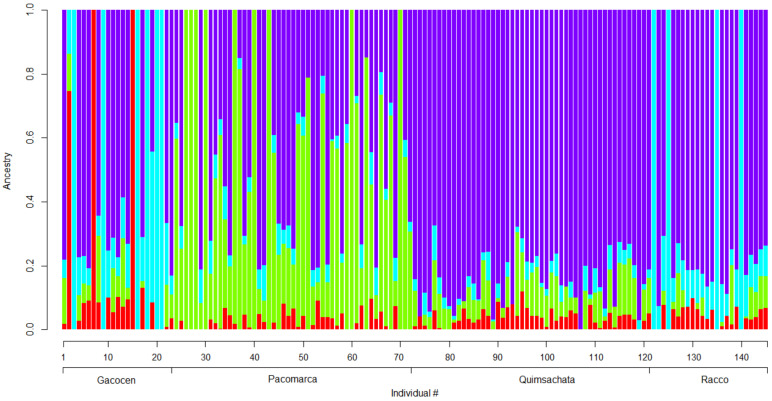
Proportions of admixture per individual for K = 4.

Genetic differentiation of the four groups of animals was based on 76,206 SNPs. The degree of divergence between populations was estimated by the FST statistics. Gacocen shows relatively less divergence with Racco (FST = 0.134) than with Pacomarca (FST = 0.156) and with Quimsachata (FST = 0.177). Similarly, Pacomarca and Quimsachata are more divergent (FST = 0.098) than Pacomarca and Racco (FST = 0.053) and Quimsachata and Racco (FST = 0.075). These differences can be visualized in Figure 6 that shows the analysis of ancestry per individual for the 145 animals considering a K = 4 and a cross-validation error CV = 0.53902.

## 4. Discussion

### 4.1. Animal Samples

Microarray development requires the identification of large numbers of SNPs, from which reliable SNPs can be identified and included in the microarray. To identify large numbers of SNPs, other research groups [17,18,19] used animal samples from different breeds and/or from different breeding selection lines [19] already showing significant phenotypic diversity and therefore assumed to harbor genetic sequence diversity. In the case of alpaca, there are only two breeds, the Huacaya and Suri, representing 80.4% and 12.2% of the population of alpacas, respectively. Interbreed, and interspecies crosses represent 7.3% [35]. In the absence of background information about the genetic diversity of the alpaca population in Peru, the selection of animal samples reared extensively and under some form of farm management was determined to be our best alternative to warrant some genetic diversity among animals. Therefore, we selected Huacaya animals from two different regions and two farms per region for SNP discovery.

### 4.2. Selection of SNPs for the Microarray

The use of 10 alpaca genomes and 300 RRLs sequenced at depths of 30× and 6×, respectively, coupled to appropriate quality filters to identify SNPs with MAFs preferentially above 0.05, allowed the identification of a large number of reliable SNPs for the microarray. The approach we used is generally similar to other approaches [17,18,19] but with a higher depth of sequencing. In this manner, 4.28 × 10^6^ SNPs were identified, from which 513,467 de novo SNPs, with the minimum quality criteria required for developing a microarray, were selected.

Here we report the final selection of 80,201 novel alpaca SNPs and the development of the first alpaca SNP microarray containing 76,508 SNPs. This microarray will be useful for animal identification, confirmation of pedigree parentage, genome-wide association studies, and eventually marker and genomic selection. We envision that this tool will facilitate the development of genetic improvement programs at a small producer level.

One aspect of our microarray development was to ensure that reliable SNPs are uniformly located at intervals between 30 to 40 kbp along the length of the genome. The average microarray interval spacing between SNPs is 26.58 ± 18.57 kbp. However, there are 12,713 SNPs located in genome fragments greater than 40 kbp, with 410 of these located in fragments greater than 100 kbp. The genome length of the 88 localized scaffolds is 1.602 Gb [5], of which 0.076 Gb do not have an SNP assigned. Likewise, the genome length of the 77,302 unlocalized scaffolds is 0.517 Gb [5], and 74,228 of these (0.124 Gb) do not have an SNP assigned. Among this latter group, 72,387 scaffolds are less than 5 kbp and 1841 larger than 5 kbp.

The total length of the genome not covered with microarray SNPs is 9.48% (Table 5). Table S3 presents the distribution of SNPs and average interval spacing per chromosome, as well as the number of unlocalized scaffolds. Since many of the large VicPac3.1 chromosome assemblies remain fragmented and incomplete, the majority of the unlocalized scaffolds with no SNPs may represent, once localized, larger chromosomal spacing gaps between SNPs. Overall, the microarray covers 90.5% of the genome length with SNPs.

Of the 76,508 SNPs, the majority of SNPs (63,795 or 83%) are localized at intervals between 10 kbp to 40 kbp, with only 55 SNPs at intervals ≥ of 200 kbp. Considering all scaffolds assigned to chromosomes, the average chromosomal density of SNPs is 39 ± 2.51 SNPs/Mb. In addition, 36,993 SNPs (52%) are located at intron or exon sequences within the span of 14,096 genes and/or annotated loci in VicPac3.1 (Table S9).

SNPs average MAFs is 0.215 ± 0.139, where 94% of the SNPs have MAFs ≥ 0.05. SNP MAFs distribution is presented in Figure S2 and MAFs per SNP in Table S9.

The genes *MC1R* (melanocortin 1 receptor) and *ASIP* (agouti signaling protein) have been found to regulate alpaca fiber color [14,36]. Other important mammalian color genes, such as tyrosinase-related protein 1 (*TYRP1*), *KIT* oncogene and *KIT* oncogene ligand (*KITLG*), are also considered regulators of fiber color. Likewise, some keratin (*KRT*) and keratin-associated protein (*KRTAP*) genes are important candidate genes for fleece and fiber quality [37]. These latter genes are found in clusters in alpaca chromosomes 12 (25 *KRT* genes) and chr16 (22 *KRT* genes and 2 *KRTAPs*). The set of 302 candidate gene SNPs included in the microarray showed an average MAF = 0.1685 ± 0.1361. Twelve of these SNPs were monomorphic, 175 showed MAFs lower than 0.01, and 187 SNPs showed MAFs between 0.1 and 0.5 in the 145 animals of the sample population. A list of these SNPs and their corresponding MAFs are presented in Table S8. With the exception of the 16 SNPs reported in the literature for these candidate genes [12,14,15], all other SNPs are novel and have now been positively identified by genotyping.

### 4.3. Performance of the Alpaca SNP Microarray

Performance of the microarray was evaluated by genotyping the group of animals of the original sample and 68 animals to assess paternity relationships. Call rate of 98.9% for both groups of animals and conversion rates of 98.8% and 89.8% were observed for 145 of the 150 animals of the original sample and the 68 animals of the paternity analysis group, respectively. The higher conversion rate obtained with the original sample was expected because these animals were used to generate the SNPs. These conversion rates are comparable to conversion rates obtained with microarrays for other domestic species [17,18,19]. In addition, it was observed that 7652 SNPs had MAFs < 0.05, and 1021 SNPs were monomorphic in the sample of 145 animals.

Parent information recorded in 30 animal pedigrees was evaluated by analyzing genomic relationships of sires, dams and progenies (trios) obtained by microarray genotyping. Among these trios, there were five sires with two half-sib progenies each and three sires with three, four and five half-sib progenies each, respectively. For this analysis, monomorphic and low genotyping rate SNPs were removed. After genotyping quality control, only 61,086 SNPs per animal were used for analysis. Calculated genomic relationships showed that 4 out of 30 sire–progeny pairs were not concordant with their sire pedigree annotation. This is equivalent to a 13% error rate, which is similar to pedigree parent annotation error rates for other species [38]. Likewise, it was observed that one dam was wrongly assigned to a progeny pedigree record and that one progeny, among the thirty progenies analyzed, had both parents, sire and dam, wrongly assigned to its pedigree record. Errors in pedigree dam annotations are rare, and errors where both parents are wrongly annotated are even rarer. Similarly, among the sets of half-sibs, there were two half-sibs wrongly assigned to a set confirming the findings of the sire–progeny comparisons. Table S4 shows sire–progeny and dam–progeny G values, and Table S5 shows half-sibs G values. These results underscore the difficulties farms encounter for the maintenance of good animal records and the utility of the microarray to correct them. It also illustrates the utility of the microarray to identify animal relatedness in the absence of record keeping.

Microarray genotyping (MG) of the original sample of animals offered the possibility to compare GBS and MG genotyping results. This analysis was based on comparing the genotype calls per animal, resulting from GBS and MG for each SNP. Only SNPs that were genotyped for both methods were compared. Results are presented in Tables S6 and S7 for ApeK1 and Pst1-Msp1 SNPs, respectively. Concordance levels of 0.93 and 0.94 were observed for the sets of ApeK1 and Pst1-Msp1 SNPs, respectively. These levels of concordance are considered good when we take into account the criteria used for GBS SNP calls based on a minimum of three sequencing reads per animal. The latter is not robust enough for heterozygote calls that depend on higher read sequence depth to ascertain the genotype.

### 4.4. Sample Population Structure

This analysis provides information about the genetic variation among this group of animals and allows evaluating genetic differences, with caution, between regions and among farms. Both the population structures and the heat maps of genomic relationships obtained separately for MG and GBS (ApeK1 and Pst1-Msp1 generated SNPs) indicate that some genetic divergence exists for some animals between the Puno region and the Cerro de Pasco region. The most differentiated group of animals is the Pacomarca group. The other three farms showed some population overlap, with the exception of some Racco animals (Figure 2). Gacocen has more genetic overlap with Pacomarca and Quimsachata farms than with Racco. Hence, it looks that Cerro de Pasco farms have acquired animals and/or used sires from the Puno region for genetic improvement. However, these observations cannot be generalized because the animal sample per farm is small and does not represent the animal population of each farm. On the other hand, the heat maps of genomic relationships among animals show a higher number of related animals between the Gacocen and Racco populations located in Cerro de Pasco than the Pacomarca and Quimsachata farms located in Puno. The coefficient of inbreeding is almost negligible, and heterozygosity is 0.27 when evaluated with MG genotypes. These values seem to be in contradiction because 0.27 heterozygosity implies 0.73 homozygosity, and therefore the inbreeding value is expected to be higher than the observed 0.004. However, inbreeding coefficient and multi-locus heterozygosity do not measure the same quantity. Inbreeding measures identity by descent or autozygous genotypes, and general homozygosity measures identity by state or allozygous genotypes [39]. In a study to assess the relationship between inbreeding coefficient (F) and multi-locus heterozygosity (MLH) [40], it was found that MLH was a poor indicator of F even in populations where inbreeding was common. These authors also indicated that their findings were consistent with other findings that did not detect a significant correlation between H and F in large, randomly mating populations [41,42] or in structured populations [43]. They further indicated that marker heterozygosity does not provide a robust estimate of genome-wide heterozygosity but only may reflect heterozygosity at linked loci.

Based on the above, the heterozygosity of 0.27 measured from the genotypes of the 145 animals is perhaps a consequence of the preferable selection of SNPs with large MAFs. In fact, microarray SNPs have average MAF = 0.215 ± 0.139 where more than 31,000 SNPs present MAFs > 0.25. Another factor to consider is that about 60 animals showed negative F values. F can only be negative if there are more heterozygous genotypes observed than expected. Hence, many of the SNPs evaluated may not be in Hardy–Weinberg equilibrium. Overall, the observed heterozygosity of our sample cannot be used as a predictor of the inbreeding coefficient observed.

The development of the alpaca 76 K SNP microarray represents an important step forward to advance genetic studies of South American camelids (SACs). The use of the microarray to genotype other SACs remains to be tested. However, it is expected that a portion of the alpaca SNPs will be conserved across camelids.

## 5. Conclusions

A large number of SNPs (4.2 × 10^6^) were discovered in a sample of 150 Huacaya alpacas. This was achieved by preparing 300 reduced representation libraries (RRLs) from separate DNA digestions with ApeK1 and double digestion with Pst1-Msp1 enzymes. Sequencing of six whole Huacaya alpaca genomes to align RRLs and the availability of genome sequences of four more animals at NCBI along with the reference alpaca genome VicPac3.1 increased the accuracy and precision of the alignment of RRLs reads to identify SNPs and flanking sequences. From the pool of identified SNPs, 76,508 were selected in two steps; the first yield SNPs at 40 kbp intervals and the second reduced the interval to 26.45 ± 18.57 kbp. These SNPs are included in the microarray and constitute the first set of SNPs that have been validated for alpaca aside from 19 SNPs reported in the research literature. Hence, a first-generation alpaca 76 K SNP microarray is now available, representing a powerful tool for genome scans and for genome-wide association studies to production traits of interest. In due time, this tool will facilitate the development of genomic selection and its use in alpaca genetic improvement programs. The work reported here represents the first microarray developed for South American camelids. Since large genomic synteny exists among camelids, the use of this microarray in other camelid species remains to be tested. In particular, it could be very useful for conservation efforts in vicuña (*Vicugna vicugna*) to monitor the genetic variation of the species and help design appropriate conservation breeding programs.

## Figures and Tables

**Table 1:** Number of animals per region and farm within the region.

Region		Number of Animals
Pasco	San Pedro de Racco	25
	GACOCEN	25
Puno	Pacomarca	50
	INIA-Quimsachata	50
Total alpacas		150

**Table 2:** Selection of the first and second sets of single nucleotide polymorphisms (SNPs) by location score.

Round	Phred Score	Genotyping Rate (GR)	Minor Allele Frequency (MAF)	Illumina Score	Length of Flanking Sequences	First Set Number of SNPs	Second Set Number of SNPs
1	>10	≥0.45	0.05–0.50	≥0.60	40	45,156	17,148
2	>10	≥0.45	0.05–0.50	≥0.60	35	1319	1876
3	>10	≥0.15	0.04–0.50	≥0.60	40	4027	6734
4	>10	≥0.15	0.04–0.50	≥0.60	35	320	628
5	>10	≥0.15	0.01–0.039	≥0.60	40	829	1821
6	>10	≥0.15	0.01–0.039	≥0.60	35	121	222
Total						51,772	28,429

**Table 3:** Number of scaffold fragment lengths containing one SNP identified in this study and included in the microarray.

Fragment Lengths in kbp	Number of Fragments Containing One SNP Identified in This Study	Number of Fragments with One SNP Included in The Microarray
≥700–800	1	1
≥600–700	0	1
≥500–600	6	5
≥400–500	3	3
≥300–400	10	10
≥200–300	29	35
≥100–200	315	375
≥90–100	210	243
≥80–90	302	366
≥70–80	541	696
≥60–70	1075	1285
≥50–60	2770	3011
≥40–50	5999	6682
≥30–40	10,848	11,069
≥20–30	21,145	19,146
≥10–20	32,282	29,070
≥0–10	4683	4510
Total	80,201	76,508

**Table 4:** Number of SNPs selected for the microarray.

Description of SNPs	Nº SNPs Selected in This Study	Final Nº SNPs Selected by Affymetrix
First set	51,772	49,282
Second set	28,429	26,924
Candidate genes	302	302
Controls		100
Duplicate controls		302
Total SNPs	80,503	76,910

**Table 5:** Coverage of the alpaca genome with SNPs included in the microarray.

Scaffolds	Number of SNPs	Number of 40 kbp Fragments	Average Interval between SNPs	Length Covered by SNPs (bp)	VicPac3.1 (bp) Length	% Length of Genome Covered with SNPs (VicPac3.1)
Localized on Chromosomes	59,297	38,165	26,992	1,525,673,735	1,602,467,523	95.21
Unassigned	17,211	12,491	25,160	393,461,101	517,133,374	76.09
Total	76,508	50,656	26,580	1,919,134,836	2,119,600,897	90.54

## Data Availability

Data sharing not applicable.

## Supplementary Materials

The following are available online at https://www.mdpi.com/2073-4425/12/2/291/s1. File S1: R scripts for SNPs selection for the microarray; File S2: R script for a heat map of genomic relationships; Figure S1: Distribution of SNPs of the first and second set of selected SNPs (80 Kb) per alpaca chromosome; Figure S2: Distribution of first and second set of selected SNPs (80 Kb) by minor allele frequency (MAF); Figure S3: Population structure using GBS results for the Pstl-Mspl SNP set; Figure S4: Population structure using GBS results for the ApeK1 SNP set; Figure S5: Heat map of genomic relationships among animals base on GBS genotyping of Pstl-Mspl generated SNPs; Figure S6: Heat map of genomic relationships among animals base on GBS genotyping of ApeK1 generated SNPs; Figure S7: Coefficient of inbreeding (F) of alpacas genotyped by GBS (ApeK1 generated SNPs); Figure S8: Coefficient of inbreeding (F) of alpacas genotyped by GBS (Pst1-Msp1 generated SNPs); Figure S9: Level of heterozygosity of alpacas genotyped by GBS (ApeK1 generated SNPs); Figure S10: Level of heterozygosity of alpacas genotyped by GBS (Pst1-Msp1generated SNPs); Table S1: Example of scoring positioning of SNPs along each 40 kbp fragment within a scaffold; Table S2: First and second set of selected SNPs (80 Kb) distributed by chromosome in VicPac3.1.; Table S3: Distribution of SNPs (76,508) present in the microarray by chromosome.; Table S4: Pedigree and genomic relationships among members of trios; Table S5: Pedigree and genomic relationships (G) among half-sibs; Table S6: Comparison between GBS genotyping of SNPs generated from ApeK1 RRLs and microarray genotyping; Table S7: Comparison between GBS genotyping of SNPs generated from Pst1-Msp1 RRLs and microarray genotyping; Table S8: MAF values for 302 SNPs located in candidate genes for fiber quality and color; Table S9: List of 80,499 SNPs generated and validated by this study.
