# Supplementary material for: Development of a 76k Alpaca (Vicugna pacos) Single Nucleotide Polymorphisms (SNPs) Microarray
Source: Genes (Basel). 2021 Feb 19;12(2):291. doi: 10.3390/genes12020291 (PMC7923280; doi:10.3390/genes12020291)
Supplement: Supplementary file 1 [file genes-12-00291-s001.zip › Figures S1 - S10 01-29-2021 submission.docx]

**Figure S1.** Distribution of SNPs of the first and second set of selected SNPs (80Kb) per alpaca Chromosome, with an average chromosome density of 40 SNPs/Mb.

**Figure S2.** Distribution of first and second set of selected SNPs (80Kb) by Minor Allele Frequency (MAF).


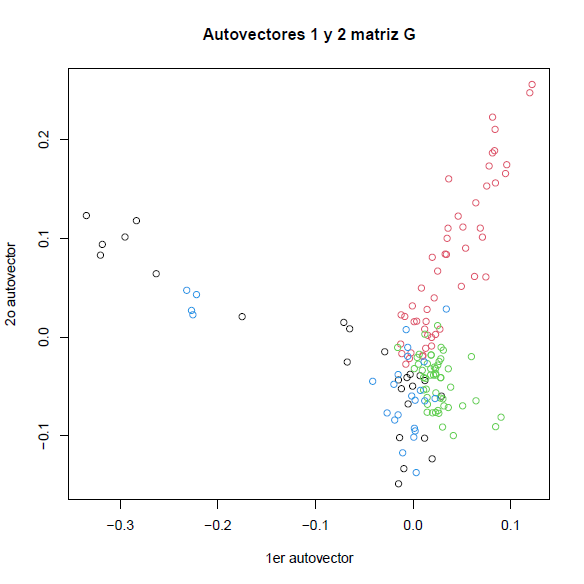


**Figure S3 (SF3).** Population structure using GBS results for the Pstl-Mspl SNP set (Pacomarca-Red, Quimsachata-green, Racco-blue and Gacocen-black).


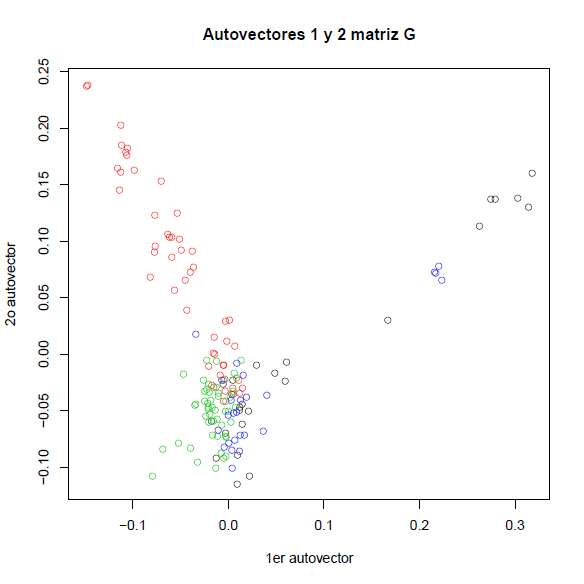


**Figure S4.** Population structure using GBS results for the ApeK1 SNP set (Pacomarca-red, Quimsachata-green, Racco-blue and Gacocen-black).


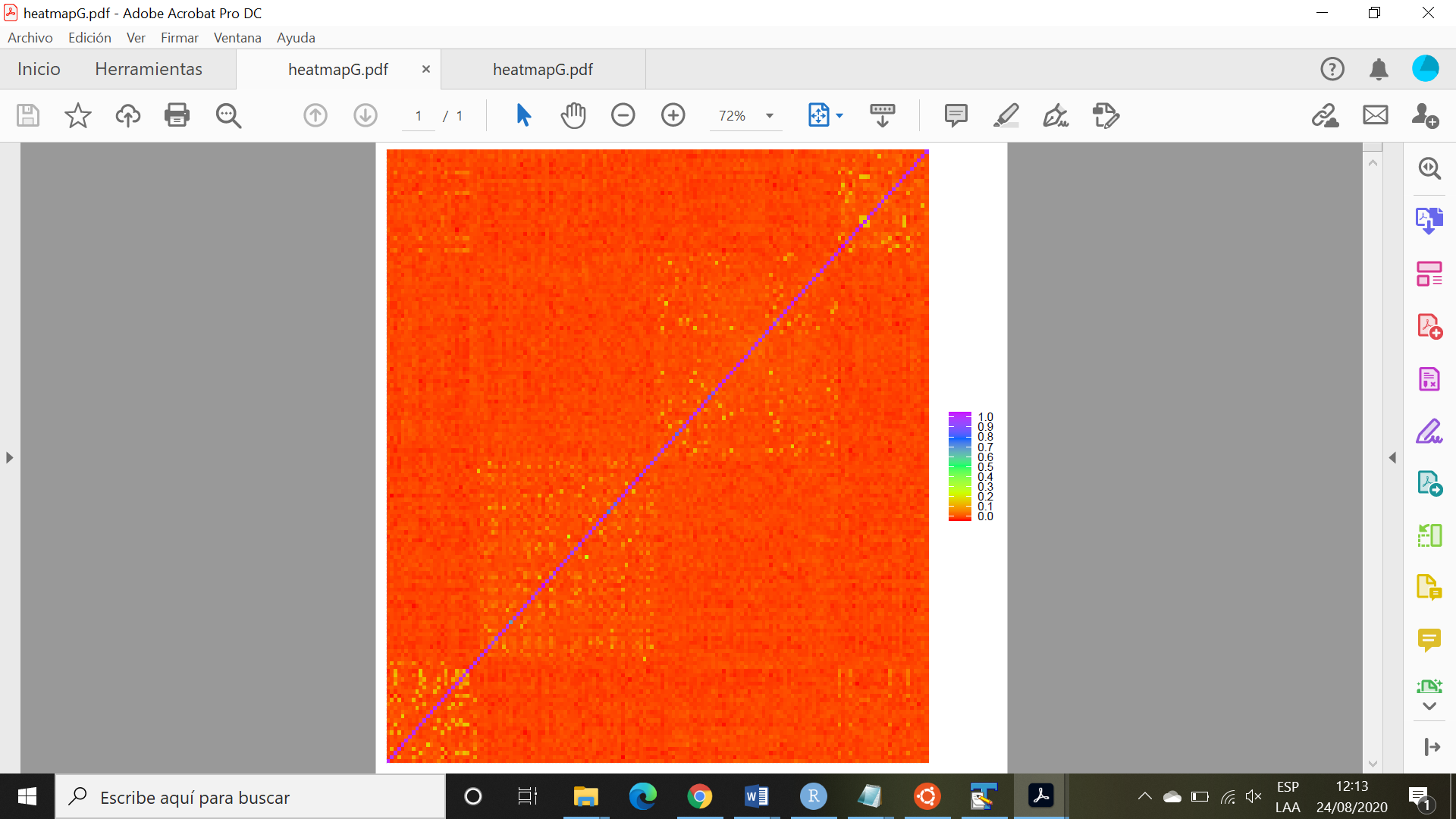


**Figure S5.** Heat map of genomic relationships among animals base on GBS genotyping of Pstl-Mspl generated SNPs (Gacocen (bottom left), Pacomarca and Quimsachata (center area) and Racco (top righ).


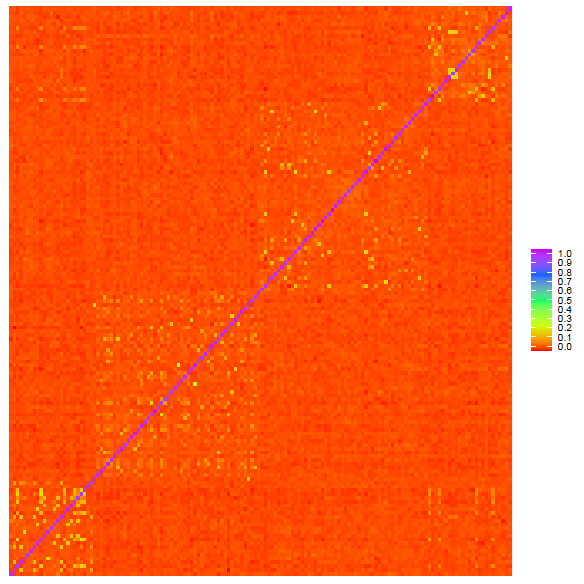


**Figure S6.** Heat map of genomic relationships among animals base on GBS genotyping for ApeK1 generated SNPs Gacocen (bottom left), Pacomarca and Quimsachata (center area) and Racco (top righ).

**Figure S7.** Coefficient of inbreeding (F) of alpacas genotyped by GBS (ApeK1 generated SNPs).

**Figure S8.** Coefficient of inbreeding (F) of alpacas genotyped by GBS (Pst1-Msp1 generated SNPs).

**Figure 9**. Level of heterozygosity of alpacas genotyped by GBS. (ApeK1 generated SNPs)

**Figure S10.** Level of heterozygosity of alpacas genotyped by GBS. (Pst1-Msp1generated SNPs).
