# Supplementary material for: Development of a 76k Alpaca (Vicugna pacos) Single Nucleotide Polymorphisms (SNPs) Microarray
Source: Genes (Basel). 2021 Feb 19;12(2):291. doi: 10.3390/genes12020291 (PMC7923280; doi:10.3390/genes12020291)
Supplement: Supplementary file 1 [file genes-12-00291-s001.zip › File S1.docx]

**File S1. R scripts for SNPs selection for the microarray**

##Datasets

#Genome assembly VicPac3.1

assembly<-read.table("Vp3_assembly.txt",header=TRUE,sep="\t")

#SNPs identified based on sequencing with Phred Score>10

q10<-read.csv("VariantCall_vp3.1_APM_q10_sort_u.csv",header=TRUE)

#Missing genotyping rate and minor allele frequency

KGD<-read.csv("APM_freq_lmiss.csv",header=TRUE)

#Illumina Score

Illumina<-read.csv("Illumina_Score.csv",header=TRUE)

#SNPs without other SNPs within flanking sequences

FSS40<-read.csv("snp_vp3.1_apm_FS_FSS40_SU_uniq.bed",header=FALSE,sep=" ")

FSS35<-read.csv("snp_vp3.1_apm_FS_FSS35_SU_uniq.bed",header=FALSE,sep=" ")

##Data management

FSS40a<-FSS40[,c(1,3)]

FSS40a_col<-c("CHROM","POS")

colnames(FSS40a)<-FSS40a_col

FSS40a$CHROM_POS<-paste(FSS40$V1,"_",FSS40$V3,sep="")

write.csv(FSS40a,"FSS40a.csv",row.names=FALSE)

FSS35a<-FSS35[,c(1,3)]

FSS35a_col<-c("CHROM","POS")

colnames(FSS35a)<-FSS35a_col

FSS35a$CHROM_POS<-paste(FSS35$V1,"_",FSS35$V3,sep="")

write.csv(FSS35a,"FSS35a.csv",row.names=FALSE)

q10_KGD<-merge(x=q10, y=KGD, by.x="X.CHROM_POS", by.y="NAME")

q10_KGD_Illumina<-merge(x=q10_KGD, y=Illumina, by.x="X.CHROM_POS", by.y="NAME")

q10_KGD_Illumina_FSS40<-merge(x=q10_KGD_Illumina, y=FSS40a, by.x="X.CHROM_POS", by.y="CHROM_POS")

q10_KGD_Illumina_FSS35<-merge(x=q10_KGD_Illumina, y=FSS35a, by.x="X.CHROM_POS", by.y="CHROM_POS")

library(dplyr)

library(ggplot2)

library(ggpubr)

library(psych)

##Rounds of selection

#Round 1

filter_CR45_MAF550_IScore60_FSS40<-subset(q10_KGD_Illumina_FSS40,q10_KGD_Illumina_FSS40$F_MISS<0.55 & q10_KGD_Illumina_FSS40$MAF>=0.05 & q10_KGD_Illumina_FSS40$MAF<=0.5 & q10_KGD_Illumina_FSS40$Final_Score>=0.6)

filter2_CR45_MAF550_IScore60_FSS40<-distinct(filter_CR45_MAF550_IScore60_FSS40, X.CHROM, POS.x, .keep_all= TRUE)

table_CR45_MAF550_IScore60_FSS40<-filter2_CR45_MAF550_IScore60_FSS40[,c(2:3,10,14)]

q10_CR45_MAF550_IScore60_FSS40_assembly<-merge(x=table_CR45_MAF550_IScore60_FSS40,y=assembly,by.x="X.CHROM",by.y="GenBank_Accn")

write.csv(q10_CR45_MAF550_IScore60_FSS40_assembly[,c("X.CHROM","POS.x","Assigned_Molecule","Assigned_Molecule_Location_Type","Sequence_Length")],"q10_CR45_MAF550_IScore60_FSS40_assembly.csv",row.names=FALSE)

q10_CR45_MAF550_IScore60_FSS40_assembly$pos4k<-(q10_CR45_MAF550_IScore60_FSS40_assembly$POS.x)/40000

q10_CR45_MAF550_IScore60_FSS40_assembly$gpos<-ceiling(q10_CR45_MAF550_IScore60_FSS40_assembly$pos4k)

q10_CR45_MAF550_IScore60_FSS40_assembly$coverage <- if_else((q10_CR45_MAF550_IScore60_FSS40_assembly$gpos*40000) < q10_CR45_MAF550_IScore60_FSS40_assembly$Sequence_Length, 40000, q10_CR45_MAF550_IScore60_FSS40_assembly$Sequence_Length-((q10_CR45_MAF550_IScore60_FSS40_assembly$gpos-1)*40000))

q10_CR45_MAF550_IScore60_FSS40_assembly$S<-(40000*(q10_CR45_MAF550_IScore60_FSS40_assembly$gpos-1))+1

q10_CR45_MAF550_IScore60_FSS40_assembly$E<-40000*(q10_CR45_MAF550_IScore60_FSS40_assembly$gpos)

q10_CR45_MAF550_IScore60_FSS40_assembly$EmS<-q10_CR45_MAF550_IScore60_FSS40_assembly$E-q10_CR45_MAF550_IScore60_FSS40_assembly$S

q10_CR45_MAF550_IScore60_FSS40_assembly$aa<-2*q10_CR45_MAF550_IScore60_FSS40_assembly$POS.x

q10_CR45_MAF550_IScore60_FSS40_assembly$EMS<-q10_CR45_MAF550_IScore60_FSS40_assembly$E+q10_CR45_MAF550_IScore60_FSS40_assembly$S

q10_CR45_MAF550_IScore60_FSS40_assembly$Score<-q10_CR45_MAF550_IScore60_FSS40_assembly$EmS-(abs(q10_CR45_MAF550_IScore60_FSS40_assembly$aa-q10_CR45_MAF550_IScore60_FSS40_assembly$EMS))

q10_CR45_MAF550_IScore60_FSS40_assembly$FSS<-"40pb"

q10_CR45_MAF550_IScore60_FSS40_assembly$Round<-1

q10_CR45_MAF550_IScore60_FSS40_assembly$f<-ceiling((q10_CR45_MAF550_IScore60_FSS40_assembly$pos4k-trunc(q10_CR45_MAF550_IScore60_FSS40_assembly$pos4k))*5)

q10_CR45_MAF550_IScore60_FSS40_assembly$find<-if_else(q10_CR45_MAF550_IScore60_FSS40_assembly$f<=3,q10_CR45_MAF550_IScore60_FSS40_assembly$f+2,q10_CR45_MAF550_IScore60_FSS40_assembly$f-2)

write.csv(q10_CR45_MAF550_IScore60_FSS40_assembly[,c("X.CHROM","POS.x","Assigned_Molecule","Assigned_Molecule_Location_Type","Sequence_Length","pos4k","gpos","coverage","S","E","EmS","aa","EMS","Score","FSS","Round","f","find")],"q10_CR45_MAF550_IScore60_FSS40_assembly_coverage.csv",row.names=FALSE)

q10_CR45_MAF550_IScore60_FSS40_assembly2<-q10_CR45_MAF550_IScore60_FSS40_assembly[order(q10_CR45_MAF550_IScore60_FSS40_assembly$X.CHROM,q10_CR45_MAF550_IScore60_FSS40_assembly$gpos,q10_CR45_MAF550_IScore60_FSS40_assembly$Score,decreasing = TRUE),]

write.csv(q10_CR45_MAF550_IScore60_FSS40_assembly2[,c("X.CHROM","POS.x","Assigned_Molecule","Assigned_Molecule_Location_Type","Sequence_Length","pos4k","gpos","coverage","S","E","EmS","aa","EMS","Score","FSS","Round","f","find")],"q10_CR45_MAF550_IScore60_FSS40_assembly2_coverage.csv",row.names=FALSE)

q10_CR45_MAF550_IScore60_FSS40_assembly3<-q10_CR45_MAF550_IScore60_FSS40_assembly2[!duplicated(q10_CR45_MAF550_IScore60_FSS40_assembly2[c("X.CHROM","gpos")]), ]

write.csv(q10_CR45_MAF550_IScore60_FSS40_assembly3[,c("X.CHROM","POS.x","Assigned_Molecule","Assigned_Molecule_Location_Type","Sequence_Length","pos4k","gpos","coverage","S","E","EmS","aa","EMS","Score","FSS","Round","f","find")],"q10_CR45_MAF550_IScore60_FSS40_assembly3_coverage.csv",row.names=FALSE)

#Round 2

filter_CR45_MAF550_IScore60_FSS35<-subset(q10_KGD_Illumina_FSS35,q10_KGD_Illumina_FSS35$F_MISS<0.55 & q10_KGD_Illumina_FSS35$MAF>=0.05 & q10_KGD_Illumina_FSS35$MAF<=0.5 & q10_KGD_Illumina_FSS35$Final_Score>=0.6)

filter2_CR45_MAF550_IScore60_FSS35<-distinct(filter_CR45_MAF550_IScore60_FSS35, X.CHROM, POS.x, .keep_all= TRUE)

table_CR45_MAF550_IScore60_FSS35<-filter2_CR45_MAF550_IScore60_FSS35[,c(2:3,10,14)]

q10_CR45_MAF550_IScore60_FSS35_assembly<-merge(x=table_CR45_MAF550_IScore60_FSS35,y=assembly,by.x="X.CHROM",by.y="GenBank_Accn")

write.csv(q10_CR45_MAF550_IScore60_FSS35_assembly[,c("X.CHROM","POS.x","Assigned_Molecule","Assigned_Molecule_Location_Type","Sequence_Length")],"q10_CR45_MAF550_IScore60_FSS35_assembly.csv",row.names=FALSE)

q10_CR45_MAF550_IScore60_FSS35_assembly$pos4k<-(q10_CR45_MAF550_IScore60_FSS35_assembly$POS.x)/40000

q10_CR45_MAF550_IScore60_FSS35_assembly$gpos<-ceiling(q10_CR45_MAF550_IScore60_FSS35_assembly$pos4k)

q10_CR45_MAF550_IScore60_FSS35_assembly$coverage <- if_else((q10_CR45_MAF550_IScore60_FSS35_assembly$gpos*40000) < q10_CR45_MAF550_IScore60_FSS35_assembly$Sequence_Length, 40000, q10_CR45_MAF550_IScore60_FSS35_assembly$Sequence_Length-((q10_CR45_MAF550_IScore60_FSS35_assembly$gpos-1)*40000))

q10_CR45_MAF550_IScore60_FSS35_assembly$S<-(40000*(q10_CR45_MAF550_IScore60_FSS35_assembly$gpos-1))+1

q10_CR45_MAF550_IScore60_FSS35_assembly$E<-40000*(q10_CR45_MAF550_IScore60_FSS35_assembly$gpos)

q10_CR45_MAF550_IScore60_FSS35_assembly$EmS<-q10_CR45_MAF550_IScore60_FSS35_assembly$E-q10_CR45_MAF550_IScore60_FSS35_assembly$S

q10_CR45_MAF550_IScore60_FSS35_assembly$aa<-2*q10_CR45_MAF550_IScore60_FSS35_assembly$POS.x

q10_CR45_MAF550_IScore60_FSS35_assembly$EMS<-q10_CR45_MAF550_IScore60_FSS35_assembly$E+q10_CR45_MAF550_IScore60_FSS35_assembly$S

q10_CR45_MAF550_IScore60_FSS35_assembly$Score<-q10_CR45_MAF550_IScore60_FSS35_assembly$EmS-(abs(q10_CR45_MAF550_IScore60_FSS35_assembly$aa-q10_CR45_MAF550_IScore60_FSS35_assembly$EMS))

q10_CR45_MAF550_IScore60_FSS35_assembly$FSS<-"35pb"

q10_CR45_MAF550_IScore60_FSS35_assembly$Round<-2

q10_CR45_MAF550_IScore60_FSS35_assembly$f<-ceiling((q10_CR45_MAF550_IScore60_FSS35_assembly$pos4k-trunc(q10_CR45_MAF550_IScore60_FSS35_assembly$pos4k))*5)

q10_CR45_MAF550_IScore60_FSS35_assembly$find<-if_else(q10_CR45_MAF550_IScore60_FSS35_assembly$f<=3,q10_CR45_MAF550_IScore60_FSS35_assembly$f+2,q10_CR45_MAF550_IScore60_FSS35_assembly$f-2)

write.csv(q10_CR45_MAF550_IScore60_FSS35_assembly[,c("X.CHROM","POS.x","Assigned_Molecule","Assigned_Molecule_Location_Type","Sequence_Length","pos4k","gpos","coverage","S","E","EmS","aa","EMS","Score","FSS","Round","f","find")],"q10_CR45_MAF550_IScore60_FSS35_assembly_coverage.csv",row.names=FALSE)

q10_CR45_MAF550_IScore60_FSS35_assembly2<-q10_CR45_MAF550_IScore60_FSS35_assembly[order(q10_CR45_MAF550_IScore60_FSS35_assembly$X.CHROM,q10_CR45_MAF550_IScore60_FSS35_assembly$gpos,q10_CR45_MAF550_IScore60_FSS35_assembly$Score,decreasing = TRUE),]

write.csv(q10_CR45_MAF550_IScore60_FSS35_assembly2[,c("X.CHROM","POS.x","Assigned_Molecule","Assigned_Molecule_Location_Type","Sequence_Length","pos4k","gpos","coverage","S","E","EmS","aa","EMS","Score","FSS","Round","f","find")],"q10_CR45_MAF550_IScore60_FSS35_assembly2_coverage.csv",row.names=FALSE)

q10_CR45_MAF550_IScore60_FSS35_assembly3<-q10_CR45_MAF550_IScore60_FSS35_assembly2[!duplicated(q10_CR45_MAF550_IScore60_FSS35_assembly2[c("X.CHROM","gpos")]), ]

write.csv(q10_CR45_MAF550_IScore60_FSS35_assembly3[,c("X.CHROM","POS.x","Assigned_Molecule","Assigned_Molecule_Location_Type","Sequence_Length","pos4k","gpos","coverage","S","E","EmS","aa","EMS","Score","FSS","Round","f","find")],"q10_CR45_MAF550_IScore60_FSS35_assembly3_coverage.csv",row.names=FALSE)

#Round 3

filter_CR15_MAF450_IScore60_FSS40<-subset(q10_KGD_Illumina_FSS40,q10_KGD_Illumina_FSS40$F_MISS<0.85 & q10_KGD_Illumina_FSS40$MAF>=0.04 & q10_KGD_Illumina_FSS40$MAF<=0.5 & q10_KGD_Illumina_FSS40$Final_Score>=0.6)

filter2_CR15_MAF450_IScore60_FSS40<-distinct(filter_CR15_MAF450_IScore60_FSS40, X.CHROM, POS.x, .keep_all= TRUE)

table_CR15_MAF450_IScore60_FSS40<-filter2_CR15_MAF450_IScore60_FSS40[,c(2:3,10,14)]

q10_CR15_MAF450_IScore60_FSS40_assembly<-merge(x=table_CR15_MAF450_IScore60_FSS40,y=assembly,by.x="X.CHROM",by.y="GenBank_Accn")

write.csv(q10_CR15_MAF450_IScore60_FSS40_assembly[,c("X.CHROM","POS.x","Assigned_Molecule","Assigned_Molecule_Location_Type","Sequence_Length")],"q10_CR15_MAF450_IScore60_FSS40_assembly.csv",row.names=FALSE)

q10_CR15_MAF450_IScore60_FSS40_assembly$pos4k<-(q10_CR15_MAF450_IScore60_FSS40_assembly$POS.x)/40000

q10_CR15_MAF450_IScore60_FSS40_assembly$gpos<-ceiling(q10_CR15_MAF450_IScore60_FSS40_assembly$pos4k)

q10_CR15_MAF450_IScore60_FSS40_assembly$coverage <- if_else((q10_CR15_MAF450_IScore60_FSS40_assembly$gpos*40000) < q10_CR15_MAF450_IScore60_FSS40_assembly$Sequence_Length, 40000, q10_CR15_MAF450_IScore60_FSS40_assembly$Sequence_Length-((q10_CR15_MAF450_IScore60_FSS40_assembly$gpos-1)*40000))

q10_CR15_MAF450_IScore60_FSS40_assembly$S<-(40000*(q10_CR15_MAF450_IScore60_FSS40_assembly$gpos-1))+1

q10_CR15_MAF450_IScore60_FSS40_assembly$E<-40000*(q10_CR15_MAF450_IScore60_FSS40_assembly$gpos)

q10_CR15_MAF450_IScore60_FSS40_assembly$EmS<-q10_CR15_MAF450_IScore60_FSS40_assembly$E-q10_CR15_MAF450_IScore60_FSS40_assembly$S

q10_CR15_MAF450_IScore60_FSS40_assembly$aa<-2*q10_CR15_MAF450_IScore60_FSS40_assembly$POS.x

q10_CR15_MAF450_IScore60_FSS40_assembly$EMS<-q10_CR15_MAF450_IScore60_FSS40_assembly$E+q10_CR15_MAF450_IScore60_FSS40_assembly$S

q10_CR15_MAF450_IScore60_FSS40_assembly$Score<-q10_CR15_MAF450_IScore60_FSS40_assembly$EmS-(abs(q10_CR15_MAF450_IScore60_FSS40_assembly$aa-q10_CR15_MAF450_IScore60_FSS40_assembly$EMS))

q10_CR15_MAF450_IScore60_FSS40_assembly$FSS<-"40pb"

q10_CR15_MAF450_IScore60_FSS40_assembly$Round<-3

q10_CR15_MAF450_IScore60_FSS40_assembly$f<-ceiling((q10_CR15_MAF450_IScore60_FSS40_assembly$pos4k-trunc(q10_CR15_MAF450_IScore60_FSS40_assembly$pos4k))*5)

q10_CR15_MAF450_IScore60_FSS40_assembly$find<-if_else(q10_CR15_MAF450_IScore60_FSS40_assembly$f<=3,q10_CR15_MAF450_IScore60_FSS40_assembly$f+2,q10_CR15_MAF450_IScore60_FSS40_assembly$f-2)

write.csv(q10_CR15_MAF450_IScore60_FSS40_assembly[,c("X.CHROM","POS.x","Assigned_Molecule","Assigned_Molecule_Location_Type","Sequence_Length","pos4k","gpos","coverage","S","E","EmS","aa","EMS","Score","FSS","Round","f","find")],"q10_CR15_MAF450_IScore60_FSS40_assembly_coverage.csv",row.names=FALSE)

q10_CR15_MAF450_IScore60_FSS40_assembly2<-q10_CR15_MAF450_IScore60_FSS40_assembly[order(q10_CR15_MAF450_IScore60_FSS40_assembly$X.CHROM,q10_CR15_MAF450_IScore60_FSS40_assembly$gpos,q10_CR15_MAF450_IScore60_FSS40_assembly$Score,decreasing = TRUE),]

write.csv(q10_CR15_MAF450_IScore60_FSS40_assembly2[,c("X.CHROM","POS.x","Assigned_Molecule","Assigned_Molecule_Location_Type","Sequence_Length","pos4k","gpos","coverage","S","E","EmS","aa","EMS","Score","FSS","Round","f","find")],"q10_CR15_MAF450_IScore60_FSS40_assembly2_coverage.csv",row.names=FALSE)

q10_CR15_MAF450_IScore60_FSS40_assembly3<-q10_CR15_MAF450_IScore60_FSS40_assembly2[!duplicated(q10_CR15_MAF450_IScore60_FSS40_assembly2[c("X.CHROM","gpos")]), ]

write.csv(q10_CR15_MAF450_IScore60_FSS40_assembly3[,c("X.CHROM","POS.x","Assigned_Molecule","Assigned_Molecule_Location_Type","Sequence_Length","pos4k","gpos","coverage","S","E","EmS","aa","EMS","Score","FSS","Round","f","find")],"q10_CR15_MAF450_IScore60_FSS40_assembly3_coverage.csv",row.names=FALSE)

#Round 4

filter_CR15_MAF450_IScore60_FSS35<-subset(q10_KGD_Illumina_FSS35,q10_KGD_Illumina_FSS35$F_MISS<0.85 & q10_KGD_Illumina_FSS35$MAF>=0.04 & q10_KGD_Illumina_FSS35$MAF<=0.5 & q10_KGD_Illumina_FSS35$Final_Score>=0.6)

filter2_CR15_MAF450_IScore60_FSS35<-distinct(filter_CR15_MAF450_IScore60_FSS35, X.CHROM, POS.x, .keep_all= TRUE)

table_CR15_MAF450_IScore60_FSS35<-filter2_CR15_MAF450_IScore60_FSS35[,c(2:3,10,14)]

q10_CR15_MAF450_IScore60_FSS35_assembly<-merge(x=table_CR15_MAF450_IScore60_FSS35,y=assembly,by.x="X.CHROM",by.y="GenBank_Accn")

write.csv(q10_CR15_MAF450_IScore60_FSS35_assembly[,c("X.CHROM","POS.x","Assigned_Molecule","Assigned_Molecule_Location_Type","Sequence_Length")],"q10_CR15_MAF450_IScore60_FSS35_assembly.csv",row.names=FALSE)

q10_CR15_MAF450_IScore60_FSS35_assembly$pos4k<-(q10_CR15_MAF450_IScore60_FSS35_assembly$POS.x)/40000

q10_CR15_MAF450_IScore60_FSS35_assembly$gpos<-ceiling(q10_CR15_MAF450_IScore60_FSS35_assembly$pos4k)

q10_CR15_MAF450_IScore60_FSS35_assembly$coverage <- if_else((q10_CR15_MAF450_IScore60_FSS35_assembly$gpos*40000) < q10_CR15_MAF450_IScore60_FSS35_assembly$Sequence_Length, 40000, q10_CR15_MAF450_IScore60_FSS35_assembly$Sequence_Length-((q10_CR15_MAF450_IScore60_FSS35_assembly$gpos-1)*40000))

q10_CR15_MAF450_IScore60_FSS35_assembly$S<-(40000*(q10_CR15_MAF450_IScore60_FSS35_assembly$gpos-1))+1

q10_CR15_MAF450_IScore60_FSS35_assembly$E<-40000*(q10_CR15_MAF450_IScore60_FSS35_assembly$gpos)

q10_CR15_MAF450_IScore60_FSS35_assembly$EmS<-q10_CR15_MAF450_IScore60_FSS35_assembly$E-q10_CR15_MAF450_IScore60_FSS35_assembly$S

q10_CR15_MAF450_IScore60_FSS35_assembly$aa<-2*q10_CR15_MAF450_IScore60_FSS35_assembly$POS.x

q10_CR15_MAF450_IScore60_FSS35_assembly$EMS<-q10_CR15_MAF450_IScore60_FSS35_assembly$E+q10_CR15_MAF450_IScore60_FSS35_assembly$S

q10_CR15_MAF450_IScore60_FSS35_assembly$Score<-q10_CR15_MAF450_IScore60_FSS35_assembly$EmS-(abs(q10_CR15_MAF450_IScore60_FSS35_assembly$aa-q10_CR15_MAF450_IScore60_FSS35_assembly$EMS))

q10_CR15_MAF450_IScore60_FSS35_assembly$FSS<-"35pb"

q10_CR15_MAF450_IScore60_FSS35_assembly$Round<-4

q10_CR15_MAF450_IScore60_FSS35_assembly$f<-ceiling((q10_CR15_MAF450_IScore60_FSS35_assembly$pos4k-trunc(q10_CR15_MAF450_IScore60_FSS35_assembly$pos4k))*5)

q10_CR15_MAF450_IScore60_FSS35_assembly$find<-if_else(q10_CR15_MAF450_IScore60_FSS35_assembly$f<=3,q10_CR15_MAF450_IScore60_FSS35_assembly$f+2,q10_CR15_MAF450_IScore60_FSS35_assembly$f-2)

write.csv(q10_CR15_MAF450_IScore60_FSS35_assembly[,c("X.CHROM","POS.x","Assigned_Molecule","Assigned_Molecule_Location_Type","Sequence_Length","pos4k","gpos","coverage","S","E","EmS","aa","EMS","Score","FSS","Round","f","find")],"q10_CR15_MAF450_IScore60_FSS35_assembly_coverage.csv",row.names=FALSE)

q10_CR15_MAF450_IScore60_FSS35_assembly2<-q10_CR15_MAF450_IScore60_FSS35_assembly[order(q10_CR15_MAF450_IScore60_FSS35_assembly$X.CHROM,q10_CR15_MAF450_IScore60_FSS35_assembly$gpos,q10_CR15_MAF450_IScore60_FSS35_assembly$Score,decreasing = TRUE),]

write.csv(q10_CR15_MAF450_IScore60_FSS35_assembly2[,c("X.CHROM","POS.x","Assigned_Molecule","Assigned_Molecule_Location_Type","Sequence_Length","pos4k","gpos","coverage","S","E","EmS","aa","EMS","Score","FSS","Round","f","find")],"q10_CR15_MAF450_IScore60_FSS35_assembly2_coverage.csv",row.names=FALSE)

q10_CR15_MAF450_IScore60_FSS35_assembly3<-q10_CR15_MAF450_IScore60_FSS35_assembly2[!duplicated(q10_CR15_MAF450_IScore60_FSS35_assembly2[c("X.CHROM","gpos")]), ]

write.csv(q10_CR15_MAF450_IScore60_FSS35_assembly3[,c("X.CHROM","POS.x","Assigned_Molecule","Assigned_Molecule_Location_Type","Sequence_Length","pos4k","gpos","coverage","S","E","EmS","aa","EMS","Score","FSS","Round","f","find")],"q10_CR15_MAF450_IScore60_FSS35_assembly3_coverage.csv",row.names=FALSE)

#Round 5

filter_CR15_MAF150_IScore60_FSS40<-subset(q10_KGD_Illumina_FSS40,q10_KGD_Illumina_FSS40$F_MISS<0.85 & q10_KGD_Illumina_FSS40$MAF>=0.01 & q10_KGD_Illumina_FSS40$MAF<=0.5 & q10_KGD_Illumina_FSS40$Final_Score>=0.6)

filter2_CR15_MAF150_IScore60_FSS40<-distinct(filter_CR15_MAF150_IScore60_FSS40, X.CHROM, POS.x, .keep_all= TRUE)

table_CR15_MAF150_IScore60_FSS40<-filter2_CR15_MAF150_IScore60_FSS40[,c(2:3,10,14)]

q10_CR15_MAF150_IScore60_FSS40_assembly<-merge(x=table_CR15_MAF150_IScore60_FSS40,y=assembly,by.x="X.CHROM",by.y="GenBank_Accn")

write.csv(q10_CR15_MAF150_IScore60_FSS40_assembly[,c("X.CHROM","POS.x","Assigned_Molecule","Assigned_Molecule_Location_Type","Sequence_Length")],"q10_CR15_MAF150_IScore60_FSS40_assembly.csv",row.names=FALSE)

q10_CR15_MAF150_IScore60_FSS40_assembly$pos4k<-(q10_CR15_MAF150_IScore60_FSS40_assembly$POS.x)/40000

q10_CR15_MAF150_IScore60_FSS40_assembly$gpos<-ceiling(q10_CR15_MAF150_IScore60_FSS40_assembly$pos4k)

q10_CR15_MAF150_IScore60_FSS40_assembly$coverage <- if_else((q10_CR15_MAF150_IScore60_FSS40_assembly$gpos*40000) < q10_CR15_MAF150_IScore60_FSS40_assembly$Sequence_Length, 40000, q10_CR15_MAF150_IScore60_FSS40_assembly$Sequence_Length-((q10_CR15_MAF150_IScore60_FSS40_assembly$gpos-1)*40000))

q10_CR15_MAF150_IScore60_FSS40_assembly$S<-(40000*(q10_CR15_MAF150_IScore60_FSS40_assembly$gpos-1))+1

q10_CR15_MAF150_IScore60_FSS40_assembly$E<-40000*(q10_CR15_MAF150_IScore60_FSS40_assembly$gpos)

q10_CR15_MAF150_IScore60_FSS40_assembly$EmS<-q10_CR15_MAF150_IScore60_FSS40_assembly$E-q10_CR15_MAF150_IScore60_FSS40_assembly$S

q10_CR15_MAF150_IScore60_FSS40_assembly$aa<-2*q10_CR15_MAF150_IScore60_FSS40_assembly$POS.x

q10_CR15_MAF150_IScore60_FSS40_assembly$EMS<-q10_CR15_MAF150_IScore60_FSS40_assembly$E+q10_CR15_MAF150_IScore60_FSS40_assembly$S

q10_CR15_MAF150_IScore60_FSS40_assembly$Score<-q10_CR15_MAF150_IScore60_FSS40_assembly$EmS-(abs(q10_CR15_MAF150_IScore60_FSS40_assembly$aa-q10_CR15_MAF150_IScore60_FSS40_assembly$EMS))

q10_CR15_MAF150_IScore60_FSS40_assembly$FSS<-"40pb"

q10_CR15_MAF150_IScore60_FSS40_assembly$Round<-5

q10_CR15_MAF150_IScore60_FSS40_assembly$f<-ceiling((q10_CR15_MAF150_IScore60_FSS40_assembly$pos4k-trunc(q10_CR15_MAF150_IScore60_FSS40_assembly$pos4k))*5)

q10_CR15_MAF150_IScore60_FSS40_assembly$find<-if_else(q10_CR15_MAF150_IScore60_FSS40_assembly$f<=3,q10_CR15_MAF150_IScore60_FSS40_assembly$f+2,q10_CR15_MAF150_IScore60_FSS40_assembly$f-2)

write.csv(q10_CR15_MAF150_IScore60_FSS40_assembly[,c("X.CHROM","POS.x","Assigned_Molecule","Assigned_Molecule_Location_Type","Sequence_Length","pos4k","gpos","coverage","S","E","EmS","aa","EMS","Score","FSS","Round","f","find")],"q10_CR15_MAF150_IScore60_FSS40_assembly_coverage.csv",row.names=FALSE)

q10_CR15_MAF150_IScore60_FSS40_assembly2<-q10_CR15_MAF150_IScore60_FSS40_assembly[order(q10_CR15_MAF150_IScore60_FSS40_assembly$X.CHROM,q10_CR15_MAF150_IScore60_FSS40_assembly$gpos,q10_CR15_MAF150_IScore60_FSS40_assembly$Score,decreasing = TRUE),]

write.csv(q10_CR15_MAF150_IScore60_FSS40_assembly2[,c("X.CHROM","POS.x","Assigned_Molecule","Assigned_Molecule_Location_Type","Sequence_Length","pos4k","gpos","coverage","S","E","EmS","aa","EMS","Score","FSS","Round","f","find")],"q10_CR15_MAF150_IScore60_FSS40_assembly2_coverage.csv",row.names=FALSE)

q10_CR15_MAF150_IScore60_FSS40_assembly3<-q10_CR15_MAF150_IScore60_FSS40_assembly2[!duplicated(q10_CR15_MAF150_IScore60_FSS40_assembly2[c("X.CHROM","gpos")]), ]

write.csv(q10_CR15_MAF150_IScore60_FSS40_assembly3[,c("X.CHROM","POS.x","Assigned_Molecule","Assigned_Molecule_Location_Type","Sequence_Length","pos4k","gpos","coverage","S","E","EmS","aa","EMS","Score","FSS","Round","f","find")],"q10_CR15_MAF150_IScore60_FSS40_assembly3_coverage.csv",row.names=FALSE)

#Round 6

filter_CR15_MAF150_IScore60_FSS35<-subset(q10_KGD_Illumina_FSS35,q10_KGD_Illumina_FSS35$F_MISS<0.85 & q10_KGD_Illumina_FSS35$MAF>=0.01 & q10_KGD_Illumina_FSS35$MAF<=0.5 & q10_KGD_Illumina_FSS35$Final_Score>=0.6)

filter2_CR15_MAF150_IScore60_FSS35<-distinct(filter_CR15_MAF150_IScore60_FSS35, X.CHROM, POS.x, .keep_all= TRUE)

table_CR15_MAF150_IScore60_FSS35<-filter2_CR15_MAF150_IScore60_FSS35[,c(2:3,10,14)]

q10_CR15_MAF150_IScore60_FSS35_assembly<-merge(x=table_CR15_MAF150_IScore60_FSS35,y=assembly,by.x="X.CHROM",by.y="GenBank_Accn")

write.csv(q10_CR15_MAF150_IScore60_FSS35_assembly[,c("X.CHROM","POS.x","Assigned_Molecule","Assigned_Molecule_Location_Type","Sequence_Length")],"q10_CR15_MAF150_IScore60_FSS35_assembly.csv",row.names=FALSE)

q10_CR15_MAF150_IScore60_FSS35_assembly$pos4k<-(q10_CR15_MAF150_IScore60_FSS35_assembly$POS.x)/40000

q10_CR15_MAF150_IScore60_FSS35_assembly$gpos<-ceiling(q10_CR15_MAF150_IScore60_FSS35_assembly$pos4k)

q10_CR15_MAF150_IScore60_FSS35_assembly$coverage <- if_else((q10_CR15_MAF150_IScore60_FSS35_assembly$gpos*40000) < q10_CR15_MAF150_IScore60_FSS35_assembly$Sequence_Length, 40000, q10_CR15_MAF150_IScore60_FSS35_assembly$Sequence_Length-((q10_CR15_MAF150_IScore60_FSS35_assembly$gpos-1)*40000))

q10_CR15_MAF150_IScore60_FSS35_assembly$S<-(40000*(q10_CR15_MAF150_IScore60_FSS35_assembly$gpos-1))+1

q10_CR15_MAF150_IScore60_FSS35_assembly$E<-40000*(q10_CR15_MAF150_IScore60_FSS35_assembly$gpos)

q10_CR15_MAF150_IScore60_FSS35_assembly$EmS<-q10_CR15_MAF150_IScore60_FSS35_assembly$E-q10_CR15_MAF150_IScore60_FSS35_assembly$S

q10_CR15_MAF150_IScore60_FSS35_assembly$aa<-2*q10_CR15_MAF150_IScore60_FSS35_assembly$POS.x

q10_CR15_MAF150_IScore60_FSS35_assembly$EMS<-q10_CR15_MAF150_IScore60_FSS35_assembly$E+q10_CR15_MAF150_IScore60_FSS35_assembly$S

q10_CR15_MAF150_IScore60_FSS35_assembly$Score<-q10_CR15_MAF150_IScore60_FSS35_assembly$EmS-(abs(q10_CR15_MAF150_IScore60_FSS35_assembly$aa-q10_CR15_MAF150_IScore60_FSS35_assembly$EMS))

q10_CR15_MAF150_IScore60_FSS35_assembly$FSS<-"35pb"

q10_CR15_MAF150_IScore60_FSS35_assembly$Round<-6

q10_CR15_MAF150_IScore60_FSS35_assembly$f<-ceiling((q10_CR15_MAF150_IScore60_FSS35_assembly$pos4k-trunc(q10_CR15_MAF150_IScore60_FSS35_assembly$pos4k))*5)

q10_CR15_MAF150_IScore60_FSS35_assembly$find<-if_else(q10_CR15_MAF150_IScore60_FSS35_assembly$f<=3,q10_CR15_MAF150_IScore60_FSS35_assembly$f+2,q10_CR15_MAF150_IScore60_FSS35_assembly$f-2)

write.csv(q10_CR15_MAF150_IScore60_FSS35_assembly[,c("X.CHROM","POS.x","Assigned_Molecule","Assigned_Molecule_Location_Type","Sequence_Length","pos4k","gpos","coverage","S","E","EmS","aa","EMS","Score","FSS","Round","f","find")],"q10_CR15_MAF150_IScore60_FSS35_assembly_coverage.csv",row.names=FALSE)

q10_CR15_MAF150_IScore60_FSS35_assembly2<-q10_CR15_MAF150_IScore60_FSS35_assembly[order(q10_CR15_MAF150_IScore60_FSS35_assembly$X.CHROM,q10_CR15_MAF150_IScore60_FSS35_assembly$gpos,q10_CR15_MAF150_IScore60_FSS35_assembly$Score,decreasing = TRUE),]

write.csv(q10_CR15_MAF150_IScore60_FSS35_assembly2[,c("X.CHROM","POS.x","Assigned_Molecule","Assigned_Molecule_Location_Type","Sequence_Length","pos4k","gpos","coverage","S","E","EmS","aa","EMS","Score","FSS","Round","f","find")],"q10_CR15_MAF150_IScore60_FSS35_assembly2_coverage.csv",row.names=FALSE)

q10_CR15_MAF150_IScore60_FSS35_assembly3<-q10_CR15_MAF150_IScore60_FSS35_assembly2[!duplicated(q10_CR15_MAF150_IScore60_FSS35_assembly2[c("X.CHROM","gpos")]), ]

write.csv(q10_CR15_MAF150_IScore60_FSS35_assembly3[,c("X.CHROM","POS.x","Assigned_Molecule","Assigned_Molecule_Location_Type","Sequence_Length","pos4k","gpos","coverage","S","E","EmS","aa","EMS","Score","FSS","Round","f","find")],"q10_CR15_MAF150_IScore60_FSS35_assembly3_coverage.csv",row.names=FALSE)

#Reports

#First set

pR1<-read.csv("q10_CR45_MAF550_IScore60_FSS40_assembly3_coverage.csv",header=TRUE)

pR2<-read.csv("q10_CR45_MAF550_IScore60_FSS35_assembly3_coverage.csv",header=TRUE)

pR3<-read.csv("q10_CR15_MAF450_IScore60_FSS40_assembly3_coverage.csv",header=TRUE)

pR4<-read.csv("q10_CR15_MAF450_IScore60_FSS35_assembly3_coverage.csv",header=TRUE)

pR5<-read.csv("q10_CR15_MAF150_IScore60_FSS40_assembly3_coverage.csv",header=TRUE)

pR6<-read.csv("q10_CR15_MAF150_IScore60_FSS35_assembly3_coverage.csv",header=TRUE)

pR<-rbind(pR1,pR2,pR3,pR4,pR5,pR6)

write.csv(pR,"pR.csv",row.names=FALSE)

FirstSet<-pR[!duplicated(pR[c("X.CHROM","gpos")]), ]

write.csv(FirstSet[,c("X.CHROM","POS.x","Assigned_Molecule","Assigned_Molecule_Location_Type","Sequence_Length","pos4k","gpos","coverage","S","E","EmS","aa","EMS","Score","FSS","Round","f","find")],"FirstSet.csv",row.names=FALSE)

#Second set

sR1<-read.csv("q10_CR45_MAF550_IScore60_FSS40_assembly2_coverage.csv",header=TRUE)

sR2<-read.csv("q10_CR45_MAF550_IScore60_FSS35_assembly2_coverage.csv",header=TRUE)

sR3<-read.csv("q10_CR15_MAF450_IScore60_FSS40_assembly2_coverage.csv",header=TRUE)

sR4<-read.csv("q10_CR15_MAF450_IScore60_FSS35_assembly2_coverage.csv",header=TRUE)

sR5<-read.csv("q10_CR15_MAF150_IScore60_FSS40_assembly2_coverage.csv",header=TRUE)

sR6<-read.csv("q10_CR15_MAF150_IScore60_FSS35_assembly2_coverage.csv",header=TRUE)

sR<-rbind(sR1,sR2,sR3,sR4,sR5,sR6)

write.csv(sR,"sR.csv",row.names=FALSE)

ssRa<-sR[!duplicated(sR[c("X.CHROM","POS.x")]), ]

write.csv(ssRa[,c("X.CHROM","POS.x","Assigned_Molecule","Assigned_Molecule_Location_Type","Sequence_Length","pos4k","gpos","coverage","S","E","EmS","aa","EMS","Score","FSS","Round","f","find")],"ssRa.csv",row.names=FALSE)

List<-FirstSet[,c("X.CHROM","gpos","find")]

ssRb<-merge(x=List,y=ssRa,by.x=c("X.CHROM","gpos","find"),by.y=c("X.CHROM","gpos","f"))

write.csv(ssRb[,c("X.CHROM","POS.x","Assigned_Molecule","Assigned_Molecule_Location_Type","Sequence_Length","pos4k","gpos","coverage","S","E","EmS","aa","EMS","Score","FSS","Round","find","find.y")],"ssRb.csv",row.names=FALSE)

ssRc<-ssRb[order(ssRb$Round,ssRb$X.CHROM,ssRb$gpos,ssRb$Score),]

write.csv(ssRc[,c("X.CHROM","POS.x","Assigned_Molecule","Assigned_Molecule_Location_Type","Sequence_Length","pos4k","gpos","coverage","S","E","EmS","aa","EMS","Score","FSS","Round","find","find.y")],"ssRc.csv",row.names=FALSE)

SecondSet<-ssRc[!duplicated(ssRc[c("X.CHROM","gpos","find")]), ]

write.csv(SecondSet,"SecondSet.csv",row.names=FALSE)
