# Supplementary material for: Development of a 76k Alpaca (Vicugna pacos) Single Nucleotide Polymorphisms (SNPs) Microarray
Source: Genes (Basel). 2021 Feb 19;12(2):291. doi: 10.3390/genes12020291 (PMC7923280; doi:10.3390/genes12020291)
Supplement: Supplementary file 1 [file genes-12-00291-s001.zip › File S2.docx]

**File S2. R script for heat map of genomic relationships**

data<-read.csv("relationship.csv",header=TRUE,sep=";")

library("ggplot2")

ggp<-ggplot(v2a, aes(x = id1, y = id2, fill = rel)) +

geom_tile() +

scale_fill_gradientn(colours=rainbow(5),breaks = c(0.00,0.10,0.20,0.30,0.40,0.50,0.60,0.70,0.80,0.90,1.00)) +

labs(fill = "") +

theme(axis.title.x=element_blank(),

axis.title.y=element_blank(),

axis.text.x=element_blank(),

axis.text.y=element_blank(),

axis.ticks.x=element_blank(),

axis.ticks.y=element_blank())

ggp

pdf('heatmap.pdf')

ggp

dev.off()
