# Supplementary material for: Development of a 76k Alpaca (Vicugna pacos) Single Nucleotide Polymorphisms (SNPs) Microarray
Source: Genes (Basel). 2021 Feb 19;12(2):291. doi: 10.3390/genes12020291 (PMC7923280; doi:10.3390/genes12020291)
Supplement: Supplementary file 1 [file genes-12-00291-s001.zip › Tables S1- S7 - 01-29-2021 submission.docx]

**Table S1.** Example of scoring positioning of SNPs along each 40 Kbp fragment within a scaffold.

| **Scaffold** | **Fragment number** | **Length of Fragment (bp)** | **SNPs per Fragment** | **Score** |
| --- | --- | --- | --- | --- |
| ABRR03000001.1 | 1 | 40,000 | 2 | **39820** |
|  |  |  |  | 30752 |
| ABRR03000001.1 | 2 | 40,000 | 4 | 11248 |
|  |  |  |  | **35814** |
|  |  |  |  | 22238 |
|  |  |  |  | 19074 |
| ABRR03000001.1 | 3 | 40,000 | 2 | **22514** |
|  |  |  |  | 22264 |
| ABRR03000001.1 | 4 | 40,000 | 2 | **2612** |
|  |  |  |  | 2408 |

**Table S1 Legend.** The fragment number column indicates the consecutive number of 40 Kbp fragments contained by scaffold ABRR03000001.1 (four 40 Kbp fragments). The SNPs per fragment column indicates the number of SNPs available to choose per 40 Kbp fragment. The Score column indicates the SNPs location score. Location scores in bold highlight the chosen SNP for that particular fragment. For the first set of SNPs each scaffold was subdivided in 40 Kbp fragments starting from the first base pair of the scaffold sequence.

**Table S2.** First and second set of selected SNPs (80Kb) distributed by chromosome in VicPac3.1.

| **Chromosome** | **Number of SNPs** | **Number of 40 Kbp**  **Fragments** | **Average of Intermarker Distance** | **StdDev of Intermarker Distance** | **Chromosomal**  **Length covered by SNPs (bp)** | **VicPac3.1 Chromosomal**  **Length (bp)** | **Chromosome length**  **Covered by SNPs (%)** |
| --- | --- | --- | --- | --- | --- | --- | --- |
| 1 | 3693 | 2416 | 27343.15 | 16053.23 | 96557607 | 101041233 | 95.56 |
| 2 | 4132 | 2829 | 29371.84 | 18814.36 | 113130620 | 121370620 | 93.21 |
| 3 | 3077 | 1992 | 27087.39 | 15922.71 | 79643794 | 83363794 | 95.54 |
| 4 | 2499 | 1578 | 26258.07 | 14878.86 | 63076945 | 65636945 | 96.10 |
| 5 | 3553 | 2316 | 27092.82 | 15244.33 | 92634254 | 96274254 | 96.22 |
| 6 | 2820 | 1800 | 26515.69 | 14775.38 | 71951714 | 74791714 | 96.20 |
| 7 | 1065 | 731 | 29256.18 | 17064.91 | 29240000 | 31168711 | 93.81 |
| 8 | 2518 | 1710 | 27900.53 | 15440.99 | 68390077 | 70270077 | 97.32 |
| 9 | 2103 | 1299 | 25547.32 | 13996.72 | 51921482 | 53761482 | 96.58 |
| 10 | 1496 | 955 | 26452.18 | 15074.95 | 38182034 | 39582034 | 96.46 |
| 11 | 2957 | 1828 | 25345.51 | 13610.08 | 73019631 | 74979631 | 97.39 |
| 12 | 1747 | 1169 | 28029.14 | 16813.58 | 46706614 | 48986614 | 95.35 |
| 13 | 2491 | 1493 | 24471.38 | 12355.45 | 59685320 | 61008235 | 97.83 |
| 14 | 2315 | 1561 | 28972.18 | 18366.23 | 62424741 | 67111318 | 93.02 |
| 15 | 1321 | 795 | 24527.24 | 12483.52 | 31772912 | 32418436 | 98.01 |
| 16 | 1664 | 963 | 23450.67 | 11767.82 | 38426118 | 39074364 | 98.34 |
| 17 | 1941 | 1162 | 24171.00 | 11320.66 | 46480000 | 46944759 | 99.01 |
| 18 | 1338 | 744 | 22344.37 | 9736.16 | 29750177 | 29910177 | 99.47 |
| 19 | 1077 | 596 | 22290.07 | 9267.70 | 23822313 | 24022313 | 99.17 |
| 20 | 1608 | 948 | 23932.14 | 11824.77 | 37920000 | 38741345 | 97.88 |
| 21 | 1240 | 727 | 23778.91 | 11378.08 | 29070537 | 29520914 | 98.47 |
| 22 | 1122 | 635 | 22747.28 | 9890.59 | 25362599 | 25522599 | 99.37 |
| 23 | 1163 | 724 | 25303.64 | 12820.82 | 28920657 | 29440657 | 98.23 |
| 24 | 751 | 455 | 24420.85 | 11778.63 | 18186407 | 18346407 | 99.13 |
| 25 | 2388 | 1444 | 25184.43 | 14055.49 | 57737896 | 60195357 | 95.92 |
| 26 | 1113 | 686 | 25123.85 | 12859.22 | 27410361 | 27987978 | 97.94 |
| 27 | 994 | 565 | 22813.30 | 9904.99 | 22600000 | 22699463 | 99.56 |
| 28 | 710 | 403 | 22762.83 | 10427.74 | 16082605 | 16162605 | 99.51 |
| 29 | 979 | 634 | 26578.24 | 14157.49 | 25358588 | 26137040 | 97.02 |
| 30 | 533 | 325 | 24564.71 | 12134.35 | 12962202 | 13130742 | 98.72 |
| 31 | 594 | 339 | 22895.98 | 10136.03 | 13522737 | 13602737 | 99.41 |
| 32 | 1042 | 565 | 21792.13 | 9073.34 | 22567807 | 22732685 | 99.27 |
| 33 | 749 | 406 | 21702.86 | 8330.38 | 16221182 | 16261182 | 99.75 |
| 34 | 881 | 544 | 25076.72 | 12789.24 | 21737801 | 22097801 | 98.37 |
| 35 | 768 | 456 | 24019.86 | 11701.03 | 18199644 | 18484027 | 98.46 |
| 36 | 187 | 122 | 27950.16 | 18305.86 | 4860638 | 5377765 | 90.38 |
| X | 1128 | 782 | 30261.25 | 20496.52 | 31242392 | 34309508 | 91.06 |
| Chromosomes Total | 61757 | 38697 | 25924.45 | 14572.56 | 1546780406 | 1602467523 | 96.52 |
| Unlocalized scaffolds | 18444 | 13090 | 23692.03 | 24584.76 | 404377893 | 517133374 | 78.20 |
| Genome total | 80201 | 51787 | 25411.06 | 17418.28 | 1951158299 | 2119600897 | 92.05 |

**Table S3.** Distribution of SNPs (76,508) present in the microarray by chromosome.

| **Chromosome** | **Number of SNPs** | **Number of 40 Kbp fragments** | **Average of Intermarker Distance** | **StdDev of Intermarker Distance** | **Chromosome**  **Length covered by SNPs (bp)** | **VicPac3.1 Chromosomal**  **Length (bp)** | **Chromosome**  **Length covered by SNPs**  **(%)** |
| --- | --- | --- | --- | --- | --- | --- | --- |
| 1 | 3585 | 2377 | 28166.87 | 17194.23 | 94997607 | 101041233 | 94.02 |
| 2 | 4027 | 2795 | 30137.68 | 19576.45 | 111770620 | 121370620 | 92.09 |
| 3 | 2960 | 1966 | 28150.53 | 16727.25 | 78616390 | 83363794 | 94.31 |
| 4 | 2409 | 1564 | 27206.95 | 15661.65 | 62526287 | 65636945 | 95.26 |
| 5 | 3443 | 2287 | 27958.41 | 16071.04 | 91474254 | 96274254 | 95.01 |
| 6 | 2709 | 1768 | 27602.15 | 15914.77 | 70671714 | 74791714 | 94.49 |
| 7 | 1031 | 720 | 30220.98 | 17900.23 | 28800000 | 31168711 | 92.40 |
| 8 | 2431 | 1687 | 28899.02 | 16354.66 | 67470077 | 70270077 | 96.02 |
| 9 | 2031 | 1286 | 26431.61 | 15000.54 | 51406823 | 53761482 | 95.62 |
| 10 | 1441 | 945 | 27461.81 | 16092.56 | 37782034 | 39582034 | 95.45 |
| 11 | 2818 | 1795 | 26585.10 | 14905.72 | 71719875 | 74979631 | 95.65 |
| 12 | 1811 | 1159 | 27038.60 | 18079.15 | 46306614 | 48986614 | 94.53 |
| 13 | 2350 | 1478 | 25918.86 | 13590.82 | 59093595 | 61008235 | 96.86 |
| 14 | 2220 | 1529 | 30186.43 | 19858.38 | 61160000 | 67111318 | 91.13 |
| 15 | 1251 | 782 | 25899.67 | 13897.13 | 31252912 | 32418436 | 96.40 |
| 16 | 1684 | 951 | 23169.37 | 14201.84 | 37984166 | 39074364 | 97.21 |
| 17 | 1846 | 1153 | 25414.90 | 12548.38 | 46120000 | 46944759 | 98.24 |
| 18 | 1264 | 736 | 23652.50 | 11281.00 | 29430177 | 29910177 | 98.40 |
| 19 | 1013 | 589 | 23698.33 | 10993.81 | 23542313 | 24022313 | 98.00 |
| 20 | 1520 | 934 | 25317.69 | 13365.25 | 37360000 | 38741345 | 96.43 |
| 21 | 1192 | 724 | 24736.45 | 12198.65 | 28950537 | 29520914 | 98.07 |
| 22 | 1043 | 626 | 24470.23 | 11664.63 | 25002599 | 25522599 | 97.96 |
| 23 | 1115 | 717 | 26367.16 | 13773.23 | 28662452 | 29440657 | 97.36 |
| 24 | 718 | 449 | 25543.25 | 12896.59 | 17946407 | 18346407 | 97.82 |
| 25 | 2233 | 1422 | 26932.57 | 15652.00 | 56857896 | 60195357 | 94.46 |
| 26 | 1056 | 676 | 26479.96 | 14449.58 | 27010361 | 27987978 | 96.51 |
| 27 | 941 | 557 | 24098.22 | 11529.09 | 22280000 | 22699463 | 98.15 |
| 28 | 664 | 394 | 24339.77 | 12600.45 | 15722605 | 16162605 | 97.28 |
| 29 | 949 | 628 | 27368.18 | 14678.90 | 25120000 | 26137040 | 96.11 |
| 30 | 511 | 321 | 25597.38 | 13293.95 | 12802202 | 13130742 | 97.50 |
| 31 | 563 | 336 | 24156.69 | 11352.00 | 13402737 | 13602737 | 98.53 |
| 32 | 974 | 556 | 23313.55 | 11436.66 | 22207807 | 22732685 | 97.69 |
| 33 | 694 | 397 | 23354.08 | 10674.67 | 15880000 | 16261182 | 97.66 |
| 34 | 834 | 530 | 26472.94 | 15045.44 | 21200000 | 22097801 | 95.94 |
| 35 | 718 | 445 | 25692.55 | 14772.80 | 17759644 | 18484027 | 96.08 |
| 36 | 168 | 119 | 31111.19 | 19620.55 | 4740638 | 5377765 | 88.15 |
| X | 1080 | 767 | 31591.58 | 21235.14 | 30642392 | 34309508 | 89.31 |
| Chromosomes total | 59297 | 38165 | 26992.36 | 15753.59 | 1525673735 | 1602467523 | 95.21 |
| Unlocalized scaffolds | 17211 | 12491 | 25159.67 | 25991.20 | 393461101 | 517133374 | 76.09 |
| Genome Total | 76508 | 50656 | 26580.09 | 18571.29 | 1919134836 | 2119600897 | 90.54 |

**Table S4.** Pedigree and genomic relationships among members of trios.

| **Progeny** | **Sire** | **G** | **Dam** | **G** |
| --- | --- | --- | --- | --- |
| P-74 | P-264 | 0.500 | P-14 | 0.511 |
| P-75 | P-256 | 0.504 | P-533 | 0.490 |
| P-76 | P-252 | 0.481 | P-295 | 0.474 |
| P-84 | P-256 | 0.504 | P-305 | 0.494 |
| P-86 | P-250 | 0.467 | P-456 | 0.483 |
| P-88 | P-263 | 0.446 | P-460 | 0.463 |
| P-92 | P-51 | 0.019* | P-529 | 0.480 |
| P-105 | P-50 | 0.482 | P-364 | 0.476 |
| P-113 | P-52 | -0.045* | P-329 | 0.072* |
| P-117 | P-260 | 0.520 | P-275 | 0.478 |
| P-120 | P-258 | 0.478 | P-337 | 0.498 |
| P-126 | P-252 | 0.478 | P-385 | 0.477 |
| P-142 | P-261 | 0.480 | P-421 | 0.475 |
| P-144 | P-255 | 0.481 | P-507 | 0.479 |
| P-237 | P-255 | 0.462 | P-489 | 0.473 |
| P-433 | P-264 | 0.517 | P-34 | 0.516 |
| P-441 | P-50 | 0.495 | P-31 | 0.490 |
| P-482 | P-260 | 0.511 | P-119 | 0.494 |
| P-547 | P-251 | 0.467 | P-395 | 0.462 |
| S-25 | S-133 | 0.499 | S-15 | 0.533 |
| S-54 | S-136 | 0.457 | S-192 | 0.473 |
| S-55 | S-133 | 0.497 | S-173 | 0.530 |
| S-56 | S-133 | 0.483 | S-105 | 0.508 |
| S-77 | S-136 | 0.107* | S-124 | 0.487 |
| S-83 | S-136 | 0.463 | S-96 | 0.481 |
| S-85 | S-136 | 0.528 | S-68 | 0.515 |
| S-89 | S-133 | 0.456 | S-97 | 0.475 |
| S-191 | S-133 | 0.108* | S-174 | 0.485 |
| S-218 | S-128 | 0.469 | S-163 | 0.500 |
| P-104 | P-252 | 0.477 | P-350 | 0.488 |

**Table S4 Legend.** The progeny, sire and dam in each line represent the recorded pedigree information for each trio. G denotes the values outside the diagonal of the genomic relationship matrix (intersection Progeny and Sire, Progeny and Dam). Asterisk represent G values that do not correspond to the pedigree information.

**Table S5.** Pedigree and genomic relationships (G) among half-sibs.

| **Sire** | **Half-sibs** | | **G** |
| --- | --- | --- | --- |
| P-255 | P-144 | P-237 | 0.216 |
| P-256 | P-75 | P-84 | 0.291 |
| P-260 | P-117 | P-482 | 0.297 |
| P-264 | P-74 | P-433 | 0.281 |
| P-50 | P-105 | P-441 | 0.240 |
| P-252 | P-76 | P-126 | 0.231 |
|  |  | P-104 | 0.268 |
|  | P-126 | P-104 | 0.245 |
| S-133 | S-25 | S-55 | 0.288 |
|  |  | S-56 | 0.259 |
|  |  | S-89 | 0.197 |
|  |  | S-191* | 0.072^§^ |
|  | S-55 | S-56 | 0.332 |
|  |  | S-89 | 0.164 |
|  |  | S-191* | 0.059^§^ |
|  | S-56 | S-89 | 0.261 |
|  |  | S-191* | 0.063^§^ |
|  | S-89 | S-191* | 0.002^§^ |
| S-136 | S-77* | S-54 | 0.052 |
|  |  | S-83 | 0.044 |
|  |  | S-85 | 0.060 |
|  | S-54 | S-83 | 0.226 |
|  |  | S-85 | 0.224 |
|  | S-83 | S-85 | 0.234 |

**Table S5 Legend:** Sire and Half-sib columns represent the recorded pedigree among animals within line or block. G column denotes the values outside the diagonal of the genomic relationship matrix (intersection progeny-sire and Half-Sibs). Asterisk denotes G values that do not correspond to the pedigree information (sire, half-sib). § sign denotes G-values that do not correspond to the pedigree information for half-sibs.

**Table S6.** Comparison between GBS genotyping of SNPs generated from ApeK1 RRLs and microarray genotyping. The average concordance between both genotyping methods was 0.93.

| **Sample** | **Number of Discordant SNPs** | **Number of Concordant SNPs** | **Number of SNPs compared** | **Concordance Rate** |
| --- | --- | --- | --- | --- |
| B95064 | 726 | 7981 | 8707 | 0.92 |
| B95065 | 759 | 9003 | 9762 | 0.92 |
| B95066 | 669 | 7449 | 8118 | 0.92 |
| B95067 | 864 | 10154 | 11018 | 0.92 |
| B95068 | 777 | 9815 | 10592 | 0.93 |
| B95069 | 861 | 8164 | 9025 | 0.90 |
| B95070 | 1105 | 13522 | 14627 | 0.92 |
| B95071 | 1124 | 16710 | 17834 | 0.94 |
| B95072 | 1187 | 16666 | 17853 | 0.93 |
| B95073 | 1221 | 20882 | 22103 | 0.94 |
| B95074 | 952 | 12993 | 13945 | 0.93 |
| B95075 | 1335 | 15374 | 16709 | 0.92 |
| B95076 | 764 | 7944 | 8708 | 0.91 |
| B95077 | 1407 | 16887 | 18294 | 0.92 |
| B95078 | 1017 | 12756 | 13773 | 0.93 |
| B95079 | 1058 | 17289 | 18347 | 0.94 |
| B95080 | 1329 | 19239 | 20568 | 0.94 |
| B95081 | 1399 | 16386 | 17785 | 0.92 |
| B95082 | 1131 | 17284 | 18415 | 0.94 |
| B95083 | 1158 | 19296 | 20454 | 0.94 |
| B95084 | 1251 | 21146 | 22397 | 0.94 |
| B95085 | 1375 | 25469 | 26844 | 0.95 |
| B95086 | 1279 | 22618 | 23897 | 0.95 |
| B95087 | 1425 | 23682 | 25107 | 0.94 |
| B95088 | 1170 | 19180 | 20350 | 0.94 |
| B95089 | 920 | 11294 | 12214 | 0.92 |
| B95090 | 1015 | 14950 | 15965 | 0.94 |
| B95091 | 1369 | 17862 | 19231 | 0.93 |
| B95092 | 1214 | 17368 | 18582 | 0.93 |
| B95093 | 1248 | 20632 | 21880 | 0.94 |
| B95094 | 990 | 12973 | 13963 | 0.93 |
| B95095 | 1532 | 21323 | 22855 | 0.93 |
| B95096 | 1168 | 18220 | 19388 | 0.94 |
| B95097 | 1007 | 12736 | 13743 | 0.93 |
| B95098 | 1562 | 20160 | 21722 | 0.93 |
| B95099 | 1304 | 19310 | 20614 | 0.94 |
| B95100 | 1017 | 15626 | 16643 | 0.94 |
| B95101 | 1172 | 18396 | 19568 | 0.94 |
| B95102 | 1137 | 12868 | 14005 | 0.92 |
| B95103 | 1167 | 18344 | 19511 | 0.94 |
| B95104 | 1163 | 18230 | 19393 | 0.94 |
| B95105 | 1512 | 20113 | 21625 | 0.93 |
| B95106 | 982 | 12232 | 13214 | 0.93 |
| B95107 | 997 | 12811 | 13808 | 0.93 |
| B95108 | 1180 | 21903 | 23083 | 0.95 |
| B95109 | 1285 | 20291 | 21576 | 0.94 |
| B95110 | 1070 | 18868 | 19938 | 0.95 |
| B95111 | 1346 | 20549 | 21895 | 0.94 |
| B95112 | 1034 | 15129 | 16163 | 0.94 |
| B95113 | 844 | 12056 | 12900 | 0.93 |
| B95114 | 1361 | 18973 | 20334 | 0.93 |
| B95115 | 1206 | 15357 | 16563 | 0.93 |
| B95116 | 1211 | 19342 | 20553 | 0.94 |
| B95117 | 1175 | 16855 | 18030 | 0.93 |
| B95118 | 1232 | 19573 | 20805 | 0.94 |
| B95119 | 1089 | 13240 | 14329 | 0.92 |
| B95120 | 1009 | 14324 | 15333 | 0.93 |
| B95121 | 1201 | 21716 | 22917 | 0.95 |
| B95122 | 1006 | 14574 | 15580 | 0.94 |
| B95123 | 1187 | 17365 | 18552 | 0.94 |
| B95124 | 1250 | 21946 | 23196 | 0.95 |
| B95125 | 1092 | 14537 | 15629 | 0.93 |
| B95126 | 1393 | 19372 | 20765 | 0.93 |
| B95127 | 957 | 11094 | 12051 | 0.92 |
| B95128 | 919 | 11976 | 12895 | 0.93 |
| B95129 | 766 | 7970 | 8736 | 0.91 |
| B95130 | 1042 | 14383 | 15425 | 0.93 |
| B95131 | 1062 | 17427 | 18489 | 0.94 |
| B95132 | 921 | 12765 | 13686 | 0.93 |
| B95133 | 1075 | 17194 | 18269 | 0.94 |
| B95134 | 1032 | 15180 | 16212 | 0.94 |
| B95135 | 706 | 8536 | 9242 | 0.92 |
| B95136 | 1117 | 16827 | 17944 | 0.94 |
| B95137 | 1272 | 21373 | 22645 | 0.94 |
| B95138 | 1039 | 14202 | 15241 | 0.93 |
| B95139 | 910 | 12271 | 13181 | 0.93 |
| B95140 | 1184 | 18774 | 19958 | 0.94 |
| B95141 | 927 | 11833 | 12760 | 0.93 |
| B95142 | 1231 | 17577 | 18808 | 0.93 |
| B95143 | 933 | 12070 | 13003 | 0.93 |
| B95144 | 1026 | 14717 | 15743 | 0.93 |
| B95145 | 1117 | 15580 | 16697 | 0.93 |
| B95146 | 1262 | 19301 | 20563 | 0.94 |
| B95147 | 836 | 9038 | 9874 | 0.92 |
| B95148 | 1074 | 15596 | 16670 | 0.94 |
| B95149 | 890 | 11741 | 12631 | 0.93 |
| B95150 | 1231 | 14758 | 15989 | 0.92 |
| B95151 | 975 | 15374 | 16349 | 0.94 |
| B95152 | 900 | 13128 | 14028 | 0.94 |
| B95153 | 1068 | 15437 | 16505 | 0.94 |
| B95154 | 1175 | 18694 | 19869 | 0.94 |
| B95155 | 1160 | 19584 | 20744 | 0.94 |
| B95156 | 1048 | 17190 | 18238 | 0.94 |
| B95157 | 1085 | 13951 | 15036 | 0.93 |
| B95158 | 1193 | 20585 | 21778 | 0.95 |
| B95159 | 877 | 10555 | 11432 | 0.92 |
| B95161 | 1002 | 13550 | 14552 | 0.93 |
| B95162 | 1220 | 20227 | 21447 | 0.94 |
| B95163 | 1122 | 17211 | 18333 | 0.94 |
| B95164 | 1110 | 16591 | 17701 | 0.94 |
| B95165 | 1245 | 16074 | 17319 | 0.93 |
| B95166 | 1216 | 20370 | 21586 | 0.94 |
| B95168 | 1285 | 21837 | 23122 | 0.94 |
| B95169 | 531 | 6002 | 6533 | 0.92 |
| B95170 | 1078 | 15230 | 16308 | 0.93 |
| B95171 | 1362 | 16596 | 17958 | 0.92 |
| B95172 | 899 | 10979 | 11878 | 0.92 |
| B95173 | 1034 | 15292 | 16326 | 0.94 |
| B95174 | 1081 | 16512 | 17593 | 0.94 |
| B95175 | 1058 | 16152 | 17210 | 0.94 |
| B95176 | 1302 | 20797 | 22099 | 0.94 |
| B95177 | 1486 | 20874 | 22360 | 0.93 |
| B95178 | 1007 | 15056 | 16063 | 0.94 |
| B95179 | 1184 | 17779 | 18963 | 0.94 |
| B95180 | 980 | 13716 | 14696 | 0.93 |
| B95182 | 866 | 11773 | 12639 | 0.93 |
| B95183 | 1304 | 20871 | 22175 | 0.94 |
| B95184 | 1586 | 22011 | 23597 | 0.93 |
| B95185 | 1262 | 21919 | 23181 | 0.95 |
| B95186 | 1373 | 17647 | 19020 | 0.93 |
| B95187 | 837 | 10851 | 11688 | 0.93 |
| B95189 | 1487 | 21968 | 23455 | 0.94 |
| B95190 | 1152 | 16285 | 17437 | 0.93 |
| B95191 | 1580 | 21729 | 23309 | 0.93 |
| B95192 | 1185 | 15525 | 16710 | 0.93 |
| B95193 | 1261 | 20144 | 21405 | 0.94 |
| B95194 | 1198 | 14587 | 15785 | 0.92 |
| B95195 | 1048 | 16544 | 17592 | 0.94 |
| B95196 | 1322 | 23435 | 24757 | 0.95 |
| B95197 | 938 | 12056 | 12994 | 0.93 |
| B95198 | 1335 | 22651 | 23986 | 0.94 |
| B95200 | 1261 | 22734 | 23995 | 0.95 |
| B95201 | 1556 | 22404 | 23960 | 0.94 |
| B95202 | 1217 | 21428 | 22645 | 0.95 |
| B95203 | 1221 | 21421 | 22642 | 0.95 |
| B95204 | 1082 | 17005 | 18087 | 0.94 |
| B95205 | 1212 | 21398 | 22610 | 0.95 |
| B95206 | 1214 | 20056 | 21270 | 0.94 |
| B95207 | 1358 | 23834 | 25192 | 0.95 |
| B95208 | 1252 | 23057 | 24309 | 0.95 |
| B95209 | 1157 | 21891 | 23048 | 0.95 |
| B95210 | 1167 | 17149 | 18316 | 0.94 |
| B95211 | 1052 | 15327 | 16379 | 0.94 |
| B95212 | 1074 | 16966 | 18040 | 0.94 |
| B95213 | 971 | 13549 | 14520 | 0.93 |

**Table S7.** Comparison between GBS genotyping of SNPs generated from Pst1-Msp1 RRLs and microarray genotyping. The average concordance between both genotyping methods was 0.94.

| **Sample** | **Number of Discordant SNPs** | **Number of Concordant SNPs** | **Number of SNPs compared** | **Concordance Rate** |
| --- | --- | --- | --- | --- |
| B95064 | 447 | 5943 | 6390 | 0.93 |
| B95065 | 397 | 5516 | 5913 | 0.93 |
| B95066 | 498 | 8232 | 8730 | 0.94 |
| B95067 | 438 | 6271 | 6709 | 0.93 |
| B95068 | 336 | 4786 | 5122 | 0.93 |
| B95069 | 449 | 4512 | 4961 | 0.91 |
| B95070 | 580 | 8620 | 9200 | 0.94 |
| B95071 | 353 | 5745 | 6098 | 0.94 |
| B95072 | 425 | 5510 | 5935 | 0.93 |
| B95073 | 124 | 952 | 1076 | 0.88 |
| B95074 | 399 | 6185 | 6584 | 0.94 |
| B95075 | 548 | 7177 | 7725 | 0.93 |
| B95076 | 374 | 4817 | 5191 | 0.93 |
| B95077 | 577 | 7271 | 7848 | 0.93 |
| B95078 | 495 | 7206 | 7701 | 0.94 |
| B95079 | 498 | 8522 | 9020 | 0.94 |
| B95080 | 543 | 8121 | 8664 | 0.94 |
| B95081 | 593 | 8216 | 8809 | 0.93 |
| B95082 | 528 | 9655 | 10183 | 0.95 |
| B95083 | 486 | 8633 | 9119 | 0.95 |
| B95084 | 402 | 5748 | 6150 | 0.93 |
| B95085 | 502 | 8248 | 8750 | 0.94 |
| B95086 | 476 | 7744 | 8220 | 0.94 |
| B95087 | 465 | 6961 | 7426 | 0.94 |
| B95088 | 342 | 5164 | 5506 | 0.94 |
| B95089 | 445 | 6715 | 7160 | 0.94 |
| B95090 | 316 | 4535 | 4851 | 0.93 |
| B95091 | 533 | 7087 | 7620 | 0.93 |
| B95092 | 375 | 4236 | 4611 | 0.92 |
| B95093 | 548 | 11002 | 11550 | 0.95 |
| B95094 | 545 | 9699 | 10244 | 0.95 |
| B95095 | 520 | 7054 | 7574 | 0.93 |
| B95096 | 490 | 8143 | 8633 | 0.94 |
| B95097 | 437 | 6913 | 7350 | 0.94 |
| B95098 | 513 | 7229 | 7742 | 0.93 |
| B95099 | 434 | 6008 | 6442 | 0.93 |
| B95100 | 154 | 1340 | 1494 | 0.90 |
| B95101 | 425 | 5551 | 5976 | 0.93 |
| B95102 | 294 | 2816 | 3110 | 0.91 |
| B95103 | 438 | 6364 | 6802 | 0.94 |
| B95104 | 497 | 9284 | 9781 | 0.95 |
| B95105 | 602 | 8676 | 9278 | 0.94 |
| B95106 | 571 | 10935 | 11506 | 0.95 |
| B95107 | 435 | 5877 | 6312 | 0.93 |
| B95108 | 500 | 8729 | 9229 | 0.95 |
| B95109 | 335 | 3901 | 4236 | 0.92 |
| B95110 | 466 | 8893 | 9359 | 0.95 |
| B95111 | 573 | 10300 | 10873 | 0.95 |
| B95112 | 436 | 6206 | 6642 | 0.93 |
| B95113 | 354 | 4847 | 5201 | 0.93 |
| B95114 | 564 | 7887 | 8451 | 0.93 |
| B95115 | 428 | 5137 | 5565 | 0.92 |
| B95116 | 457 | 6904 | 7361 | 0.94 |
| B95117 | 538 | 8177 | 8715 | 0.94 |
| B95118 | 399 | 5366 | 5765 | 0.93 |
| B95119 | 220 | 2437 | 2657 | 0.92 |
| B95120 | 369 | 4627 | 4996 | 0.93 |
| B95121 | 484 | 8323 | 8807 | 0.95 |
| B95122 | 499 | 8781 | 9280 | 0.95 |
| B95123 | 494 | 6388 | 6882 | 0.93 |
| B95124 | 541 | 9627 | 10168 | 0.95 |
| B95125 | 619 | 10285 | 10904 | 0.94 |
| B95126 | 562 | 8412 | 8974 | 0.94 |
| B95127 | 495 | 7644 | 8139 | 0.94 |
| B95128 | 282 | 3033 | 3315 | 0.91 |
| B95129 | 310 | 3179 | 3489 | 0.91 |
| B95130 | 463 | 6920 | 7383 | 0.94 |
| B95131 | 429 | 6497 | 6926 | 0.94 |
| B95132 | 451 | 6520 | 6971 | 0.94 |
| B95133 | 527 | 9049 | 9576 | 0.95 |
| B95134 | 538 | 9434 | 9972 | 0.95 |
| B95135 | 475 | 7799 | 8274 | 0.94 |
| B95136 | 514 | 8710 | 9224 | 0.94 |
| B95137 | 622 | 8478 | 9100 | 0.93 |
| B95138 | 522 | 9517 | 10039 | 0.95 |
| B95139 | 534 | 10194 | 10728 | 0.95 |
| B95140 | 597 | 9603 | 10200 | 0.94 |
| B95141 | 539 | 9443 | 9982 | 0.95 |
| B95142 | 535 | 9152 | 9687 | 0.94 |
| B95143 | 397 | 5592 | 5989 | 0.93 |
| B95144 | 370 | 4757 | 5127 | 0.93 |
| B95145 | 532 | 9365 | 9897 | 0.95 |
| B95146 | 401 | 5508 | 5909 | 0.93 |
| B95147 | 478 | 6355 | 6833 | 0.93 |
| B95148 | 518 | 10494 | 11012 | 0.95 |
| B95149 | 552 | 10119 | 10671 | 0.95 |
| B95150 | 678 | 10279 | 10957 | 0.94 |
| B95151 | 431 | 9074 | 9505 | 0.95 |
| B95152 | 394 | 6337 | 6731 | 0.94 |
| B95153 | 516 | 9730 | 10246 | 0.95 |
| B95154 | 416 | 6143 | 6559 | 0.94 |
| B95155 | 381 | 6401 | 6782 | 0.94 |
| B95156 | 494 | 8502 | 8996 | 0.95 |
| B95157 | 519 | 8945 | 9464 | 0.95 |
| B95158 | 553 | 9964 | 10517 | 0.95 |
| B95159 | 564 | 10123 | 10687 | 0.95 |
| B95161 | 531 | 10229 | 10760 | 0.95 |
| B95162 | 596 | 11011 | 11607 | 0.95 |
| B95163 | 612 | 10564 | 11176 | 0.95 |
| B95164 | 573 | 10341 | 10914 | 0.95 |
| B95165 | 647 | 10791 | 11438 | 0.94 |
| B95166 | 568 | 11313 | 11881 | 0.95 |
| B95168 | 560 | 11115 | 11675 | 0.95 |
| B95169 | 453 | 8483 | 8936 | 0.95 |
| B95170 | 567 | 11173 | 11740 | 0.95 |
| B95171 | 709 | 10952 | 11661 | 0.94 |
| B95172 | 597 | 10353 | 10950 | 0.95 |
| B95173 | 508 | 9077 | 9585 | 0.95 |
| B95174 | 426 | 7235 | 7661 | 0.94 |
| B95175 | 560 | 11068 | 11628 | 0.95 |
| B95176 | 601 | 11012 | 11613 | 0.95 |
| B95177 | 627 | 11031 | 11658 | 0.95 |
| B95178 | 500 | 9832 | 10332 | 0.95 |
| B95179 | 633 | 10851 | 11484 | 0.94 |
| B95180 | 607 | 10250 | 10857 | 0.94 |
| B95182 | 513 | 9943 | 10456 | 0.95 |
| B95183 | 628 | 11070 | 11698 | 0.95 |
| B95184 | 658 | 10214 | 10872 | 0.94 |
| B95185 | 548 | 10549 | 11097 | 0.95 |
| B95186 | 730 | 10669 | 11399 | 0.94 |
| B95187 | 547 | 10522 | 11069 | 0.95 |
| B95189 | 552 | 8809 | 9361 | 0.94 |
| B95190 | 618 | 10953 | 11571 | 0.95 |
| B95191 | 660 | 10188 | 10848 | 0.94 |
| B95192 | 619 | 10847 | 11466 | 0.95 |
| B95193 | 548 | 10511 | 11059 | 0.95 |
| B95194 | 611 | 10268 | 10879 | 0.94 |
| B95195 | 576 | 10208 | 10784 | 0.95 |
| B95196 | 542 | 10554 | 11096 | 0.95 |
| B95197 | 546 | 10664 | 11210 | 0.95 |
| B95198 | 571 | 10931 | 11502 | 0.95 |
| B95200 | 587 | 10993 | 11580 | 0.95 |
| B95201 | 644 | 9273 | 9917 | 0.94 |
| B95202 | 555 | 10668 | 11223 | 0.95 |
| B95203 | 554 | 10130 | 10684 | 0.95 |
| B95204 | 493 | 8333 | 8826 | 0.94 |
| B95205 | 533 | 11277 | 11810 | 0.95 |
| B95206 | 580 | 10884 | 11464 | 0.95 |
| B95207 | 586 | 10545 | 11131 | 0.95 |
| B95208 | 586 | 11069 | 11655 | 0.95 |
| B95209 | 517 | 10655 | 11172 | 0.95 |
| B95210 | 526 | 10368 | 10894 | 0.95 |
| B95211 | 562 | 10397 | 10959 | 0.95 |
| B95212 | 511 | 9556 | 10067 | 0.95 |
| B95213 | 491 | 9545 | 10036 | 0.95 |
